# PEP725: 15 years of driving European and global phenology science

**DOI:** 10.1111/nph.70869

**Published:** 2026-01-22

**Authors:** Barbara Templ, Helfried Scheifinger, Isabella Ostovary, Markus Ungersböck, Hans Ressl

**Affiliations:** ^1^ Agroscope, Agroecology and Environment Reckenholzstrasse 191 Zürich 8046 Switzerland; ^2^ PEP725 Management GeoSphere Austria Hohe Warte 38 Vienna 1190 Austria; ^3^ ETH Zurich, Forest Ecology Universitätstrasse 16 Zürich 8092 Switzerland

**Keywords:** Europe, open science, PEP725 database, phenology observations, scientific impact, utilization

## Abstract

Phenology – the timing of seasonal biological events – is a sensitive indicator of climate change and ecosystem dynamics. Long‐term, broad‐scale phenological data are crucial for understanding and predicting plant responses to environmental change. However, until the mid‐2000s, European phenological observations were scattered across national networks, limiting large‐scale analyses. In response, the Pan European Phenology (PEP725) database was established 15 years ago as an open‐access, reference‐grade infrastructure for plant phenology data. PEP725 unifies observations from over 30 countries, compiled from 1868 through the present, with all records standardized to a common protocol. The database now contains more than 13 million phenological records for *c.* 265 plant species across 46 phenophases, making it the world's largest repository of ground‐based plant phenology data. We highlight key scientific insights and cross‐sector applications enabled by the dataset, and share technical lessons learned. Looking ahead, we outline a roadmap for PEP725's evolution – including new data contributions, technological upgrades, global integration, and community engagement – to ensure it remains a vibrant, open community resource driving phenology science forward. We invite the plant science community to utilize, contribute to, and further *cocreate* this phenological data platform.

## Zusammenfassung

Phänologie – die zeitliche Abfolge saisonaler biologischer Ereignisse – ist ein empfindlicher Indikator für Klimaänderungen und Ökosystemdynamiken. Langjährige, großräumige phänologische Datensätze sind entscheidend, um Pflanzenreaktionen auf Umweltveränderungen zu verstehen und vorherzusagen. Bis Mitte der 2000er‐Jahre waren europäische Phänologiebeobachtungen jedoch über nationale Netzwerke verstreut, was grosskalige Analysen erheblich einschränkte. Als Antwort darauf wurde vor 15 Jahren die Pan European Phenology Database (PEP725) als offene, referenzfähige Infrastruktur für pflanzenphänologische Daten etabliert. PEP725 vereint Beobachtungen aus über 30 Ländern seit 1868 bis heute – alle standardisiert nach einem gemeinsamen Protokoll. Die Datenbank umfasst inzwischen mehr als 13 Millionen phänologische Beobachtungen für rund 265 Pflanzenarten über 46 Phänophasen hinweg und ist damit das weltweit größte Archiv bodenbasierter Pflanzenphänologie. Wir stellen zentrale wissenschaftliche Erkenntnisse und Anwendungen vor, die durch diesen Datensatz ermöglicht wurden, und teilen technische Erfahrungen aus dem Plattformbetrieb. Zudem skizzieren wir einen Fahrplan für die Weiterentwicklung von PEP725 – einschließlich neuer Datenbeiträge, technologischer Modernisierungen, globaler Integration und Community‐Engagement – um sicherzustellen, dass die Infrastruktur auch zukünftig ein lebendiges, offenes Gemeinschaftswerk bleibt, das die Phänologieforschung nachhaltig voranbringt. Wir laden die Pflanzenwissenschafts‐Community ein, PEP725 zu nutzen, weiter auszubauen und gemeinsam mitzugestalten.

**Table:** 

	Content	
	Summary	717
I.	Introduction	717
II.	Scientific impact of the PEP725 database	719
III.	Utilisation of the PEP725 database in diverse scientific studies	722
IV.	Future directions: Coordinated Progress for the phenology community	725
V.	Conclusion	729
	Acknowledgements	730
	References	730

## I. Introduction

Phenology, the recurring life‐cycle events of plants (such as leaf‐out, flowering, and fruiting), has gained prominence as a key bio‐indicator of environmental change. Shifts in phenological timing are among the *clearest signals* of climate warming impacts on ecosystems (Badeck *et al*., 2004; Menzel *et al*. 2006). Earlier spring flowering and leaf unfolding, longer growing seasons, and altered reproductive timing have been documented world‐wide, with implications for biodiversity, agriculture, and climate feedbacks. Recognizing these patterns, the IPCC report (Rosenzweig *et al*., 2007) highlighted long‐term phenological observations as vital evidence of climate change effects (Solomon *et al*., 2007). In Europe, numerous phenology monitoring networks – many relying on volunteer observers – have collected observations for decades or even centuries (Templ *et al*., 2018). By the early 2000s, however, these data remained fragmented in separate national databases, hindering continental‐scale analyses. Researchers lacked a unified, accessible data platform to ask large‐scale questions, such as *Are phenological trends consistent across Europe? How do species and regions differ in their responses to warming?*

**Fig. 1:**
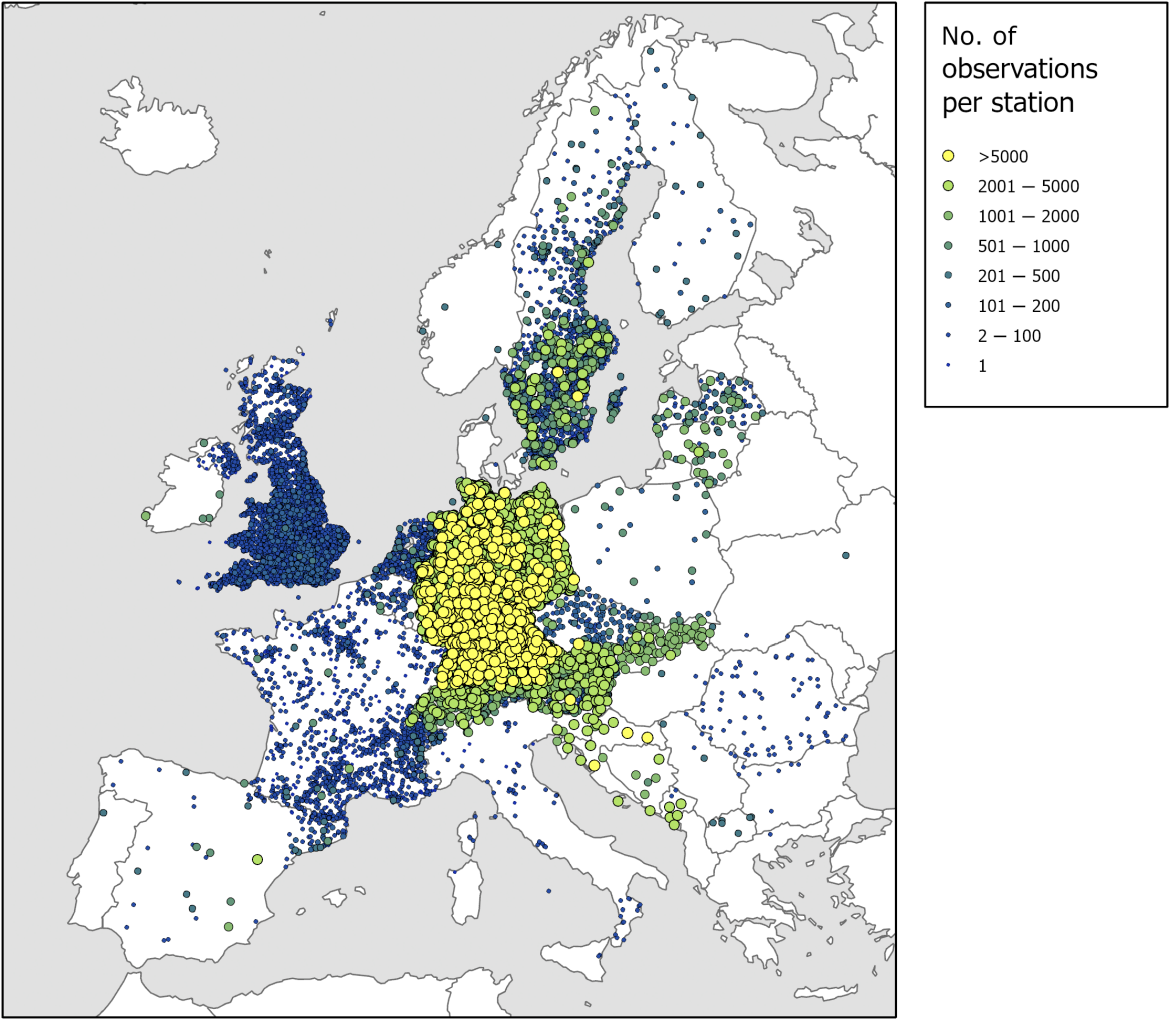
Geographic distribution of phenological observation sites across Europe captured in the PEP725 database. Stations are indicated by a colored circle; the larger the circle and the more green and yellow, the more data have been collected at the station. The amount of data usually corresponds with the length of the time series. Phenological stations cover most of Europe, from the Mediterranean to the arctic and from the Atlantic in the west to Ukraine in the east. The data density is highly variable across Europe. Countries with the highest data density and longest time series are Germany, Switzerland, Austria, Czech Republic, Slovakia, Slovenia, Croatia, and Sweden. PEP725 currently contains over 13 million phenological observations from > 20 000 locations, spanning 1775 to the present. Basic parameters of all national contributors, including temporal coverage, station and observation numbers are summarized in Supporting Information Table S2.

**Fig. 2:**
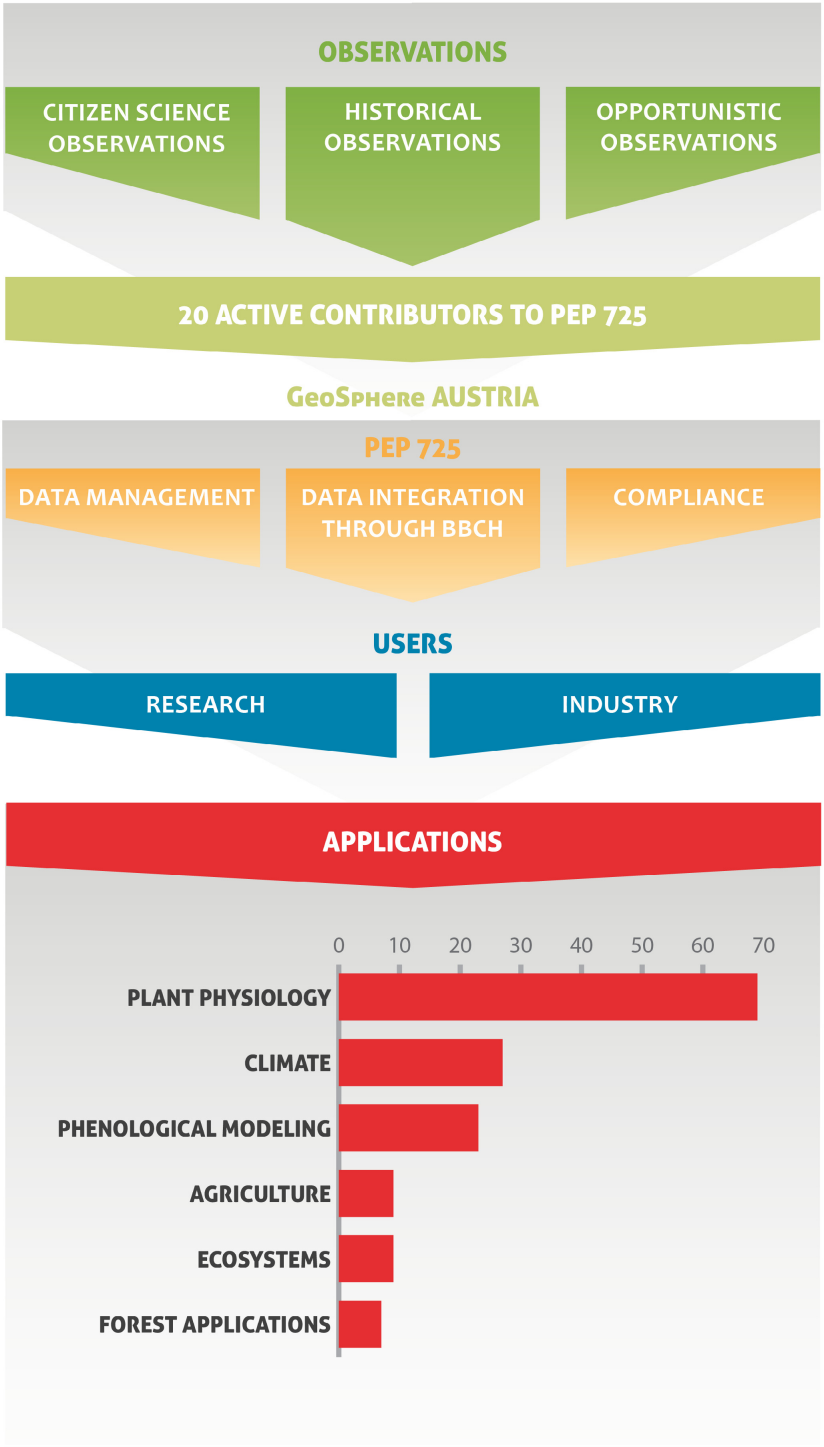
Flowchart depicting the data collection and access mechanisms and how PEP725's open‐access model supports global collaboration. Open Access to data (public access and licensing), ensuring that researchers, policymakers, and other stakeholders can freely access, utilize, and contribute to the growing repository of phenological data. This model fosters transparency, reproducibility, and widespread usage of phenological datasets, thereby enabling informed decision‐making and the advancement of scientific research across disciplines (plant physiology, remote sensing, phenological modelling, agricultural applications, ecosystems, and forest applications). Publications (*n* = 140) were counted multiple times if they covered multiple disciplines.

PEP725 was conceived to fill this gap by aggregating Europe's phenological heritage into a single open database (Templ *et al*., 2018). Initiated through the COST Action 725 ‘Establishing a European phenological data platform for climatological applications’, the project set out to compile and standardize phenology records from as many countries as possible and to make them freely available for science and education (Nekovar *et al*., 2008; Koch *et al*., 2009a,b). The underlying philosophy was to lower data access barriers; thus, PEP725 would empower researchers, educators, and other stakeholders to utilize phenology data broadly, sparking discoveries and applications. In the 15 years since its inception, PEP725 has become a cornerstone infrastructure for phenological research in Europe and beyond (Figs 1, 2). This article provides a comprehensive overview of PEP725 as a community resource, from its motivation and design to its scientific impacts and future directions (Tables 1, 2), celebrating a decade and a half of progress and looking ahead to the opportunities on the horizon.

**Table 1:** Scientific impacts enabled by the PEP725 database.

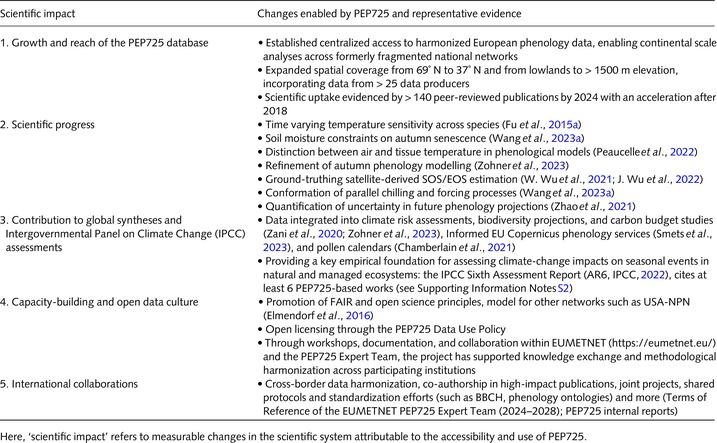

**Table 2:** Overview of scientific utilisation of the PEP725 dataset across research areas.

Research area	Impact enabled by PEP725	Representative studies
1. Plant physiology	Enabled detection of temperature, precipitation, and photoperiod drivers in spring/autumn phenology, supported new physiological hypotheses	Piao *et al*. (2015); Ettinger *et al*. (2020); Liu *et al*. (2020); Meng *et al*. (2021); Wang *et al*. (2020); Zohner *et al*. (2020, 2023)
2. Remote sensing	Provided ground truth for LSP‐GP validation, revealed LSP‐GP mismatch patterns and inspired refinement of VI‐based metrics.	Rodriguez‐Galiano *et al*. (2015); Tian *et al*. (2021); W. Wu *et al*. (2021); J. Wu *et al*. (2022); Liu *et al*. (2022); Ferrara *et al*. (2023); Yin *et al*. (2024); Zheng *et al*. (2024)
3. Phenological modeling	Enabled empirical vs process‐based model comparisons, refined climate‐phenology interaction modeling, supported machine learning applications	Xu *et al*. (2018); Olsson & Jönsson (2014); Piao *et al*. (2015); Wang *et al*. (2020); Wang *et al*. (2022); Gao *et al*. (2023); Meier & Bigler (2023)
4. Ecosystem studies	Informed ecosystem functioning, biodiversity‐phenology links, and nutrient/carbon cycles, fed into Essential Biodiversity Variables (EBV) frameworks	Kissling *et al*. (2018); Mellert *et al*. (2018); Nelson *et al*. (2018); Stucky *et al*. (2018); Gottschall *et al*. (2022)
5. Future climate scenarios	Allowed projections under future warming, water stress, urban heat, improved multi‐seasonal phenological sensitivity modelin	Jochner *et al*. (2016); Wohlfahrt *et al*. (2019); Zani *et al*. (2020); Chamberlain *et al*. (2021); Zhao *et al*. (2021)
6. Agricultural applications	Enhanced sowing/harvest timing models, supported frost risk assessment and irrigation optimization strategies	van Bussel *et al*. (2015); Fraga *et al*. (2016); Martínez‐Lüscher *et al*. (2016); Unterberger *et al*. (2018); Yang *et al*. (2022); S. Qiao *et al*. (2023)
7. Forest applications	Contributed to ozone and pollution response studies, supported forest productivity models and phenology‐climate calibration	Crabbe *et al*. (2016); Cailleret *et al*. (2018); Nölte *et al*. (2020); de Wergifosse *et al*. (2020); Xia *et al*. (2022); Vaglio Laurin *et al*. (2024)
8. Phenological databases	Anchored the development of high‐resolution and long‐term phenology databases, supported standardization via ontologies	Kissling *et al*. (2018); Stucky *et al*. (2018); Renner & Chmielewski (2021); Izquierdo‐Verdiguier & Zurita‐Milla (2024); Templ (2025)

This table synthesizes how the PEP725 has enabled concrete scientific impacts across a range of disciplines. ‘Impact’ is understood here as concrete applications of PEP725 that extend beyond data availability.

A complete list of acronyms used throughout the manuscript is provided in Supporting Information Table S1.

### 1. Motivation, history, and open science philosophy

PEP725 officially launched in 2010, building on groundwork laid by the COST725 Action (2004–2007), which fostered a pan‐European coordination of phenology observations (Fig. 1). The motivation was clear: climate change is not confined by national borders and understanding its effects on phenology requires data that extend across climatic, geographical, and political boundaries. No single country's records could capture the full spatial variability of European phenological patterns; thus, a coordinated, continental approach was essential.

Under the leadership of the Zentralanstalt für Meteorologie und Geodynamik (ZAMG, today called GeoSphere Austria) in Austria, and with support from the European meteorological community (EUMETNET), PEP725 invited national weather services and other phenology data holders to contribute their historical and ongoing records to a shared European database (Templ *et al*., 2018). An explicit open data policy was adopted from the start: all partners agreed that, once integrated, data should be freely available with proper citation for scientific and educational use – a major step away from earlier practices that required case‐by‐case permission or restricted reuse.

A critical challenge was to harmonize data collected under differing national protocols. Each network has its own species lists, observation methods, and phenophase definitions. Through sustained collaboration, PEP725 developed a common coding scheme and standardized phenophase definitions, largely aligned with the BBCH scale (Meier, 2018). Today, all records are classified using this shared system, ensuring that a ‘leaf unfolding’ or ‘flowering’ event carries the same meaning from Spain to Sweden. Harmonization involved mapping dozens of locally defined growth stages to *c*. 46 standardized phenophases and reconciling taxonomic names (including cultivars and regional variants) into a consistent structure. The result is a fully interoperable dataset that enables cross‐country comparisons and continental synthesis (Ressl *et al*., 2025). Another cornerstone of PEP725 is its integration of citizen science and historical observations (Fig. 2). Many European phenology programs – such as those in Germany and Austria – owe their longevity to dedicated volunteer observers whose records date back to the 1950s, with some digitized series reaching as far as 1775. PEP725 aggregates and preserves these unique time series while ensuring that data originators receive full recognition. This acknowledgement, combined with open access, has helped sustain both public engagement and institutional participation.

At present, PEP725 integrates observations from 41 phenological networks, 19 of which contributed new or updated datasets between 2020 and 2025 (details in Table S2). Fig. 2 provides a schematic overview of how these heterogeneous data streams enter and pass through the PEP725 pipeline. Data arrive via three principal channels: (1) citizen‐science observations submitted through national monitoring schemes, (2) historical observations digitized from archival sources, and (3) opportunistic citizen science observations – collected via mobile apps alongside formal monitoring schemes. For example, national initiatives such as the UK's Nature's Calendar allow submissions about species beyond their monitoring schemes; phenological datasets from botanical gardens or local research projects often collect records according to guidelines of national monitoring schemes despite not belonging to them. GeoSphere Austria coordinates data ingestion and quality management, including checks for completeness, spatial plausibility, logical consistency, and compliance with network metadata. All datasets are harmonized using BBCH growth‐stage codes before public release, ensuring adherence to shared structural and semantic standards.

PEP725's open‐access policy is implemented through its public web portal (http://www.pep725.eu). User uptake and reach are reflected in the annual number of new registrations (Fig. S1) and the geographic distribution of active users (Fig. S2), which highlights strong engagement from Europe and Asia. After a simple registration, which serves only to track usage and ensure proper citation, users can freely download standardized CSV files by species, country, or year. For example, all *Betula pendula* (silver birch) observations from Germany can be retrieved in one step. The Data Use Policy formalizes this ‘open‐for‐all’ model while requesting users to acknowledge data contributors, maintaining fairness and goodwill among participating institutions and volunteers.

This workflow from local observation to harmonized public dataset is summarized in Fig. 2, which illustrates how data streams converge through quality control into an openly accessible infrastructure. This approach embodies FAIR and Open Science principles (Wilkinson *et al*., 2016; Templ, 2025), promoting findable, accessible, interoperable, and reusable data. By combining transparency in processing, standardized metadata, and unrestricted licensing, PEP725 has become a reference infrastructure for phenology, enabling reproducible research and interdisciplinary applications across plant physiology, remote sensing, ecosystem and agricultural modeling, and forest studies.

## II. Scientific impact of the PEP725 database

The PEP725 database has evolved into a critical research infrastructure that supports a wide spectrum of scientific domains. Table 1 provides an overview of the main areas based on the literature (see more details about it in Methods S1; Table S3), where PEP725 has driven measurable scientific impact.

### 1. Growth and reach of the resource

Since its inception in 2010, the PEP725 database has evolved into one of the most comprehensive and frequently used repositories of ground phenological observations. The database integrates data from over 25 national networks and > 8000 observation sites, comprising more than 13 million site‐year records across a latitudinal span from 37° to 69° N and longitudes from −10° to 30° E (Fig. 1).

Community uptake has expanded steadily. Registered users increased fivefold within the last decade from *c*. 500 in 2015 to > 2500 in 2024 and annual data downloads have exceeded 25 000 datasets per year in 2023–2024. Access statistics from the PEP725 web portal show a broadening of the user base: roughly 40% of downloads now originate outside Europe, with increasing participation from Asia and North America (see Figs S1, S2).

Geographic coverage has also widened through continuous integration of new and digitized national datasets. Since 2018, additional observations have been incorporated from France, Norway, Ukraine, the Baltic region, and parts of the Iberian Peninsula, extending the database's representativeness in both climatic and biogeographical space.

Scientific visibility mirrors this expansion. By 2024, 140 peer‐reviewed publications are based on PEP725, with an average annual growth rate of *c*. 15% per year since 2018. The dataset is increasingly referenced beyond Europe, featuring in global meta‐analyses and Earth system model evaluations.

### 2. Scientific breakthroughs

Several methodological innovations have emerged through the dataset's temporal depth, continental spatial coverage, and multi‐species replication. Ground truth datasets from PEP725 have been critical for developing and refining phenological models, especially in incorporating chilling and forcing interactions (Fu *et al*., 2015b; Wang *et al*., 2023b). Multi‐model comparisons (Meier *et al*., 2024; Meier & Bigler, 2023) have used PEP725 as a reference to assess parameter sensitivity across climate scenarios, especially in spring and autumn onset estimation.

New statistical tools and calibration methods for matching ground phenology with land‐surface phenology (LSP) have emerged, using PEP725 as a benchmark (Tian *et al*., 2021; W. Wu *et al*., 2021; J. Wu *et al*., 2022). For instance, studies introduced refined inflection‐point detection on VI (Vegetation Index) trajectories (Ferrara *et al*., 2023), and new indices such as Photochemical Reflectance Index and Solar‐Induced Fluorescence (SIF) have been validated against PEP725 for improved SOS/EOS detection (Liu *et al*., 2022).

The processes governing end of season phenological phases, such as leaf senescence and abscission, remain less studied than spring phases. However, recent work has substantially advanced understanding of the environmental and physiological mechanisms controlling the onset of autumn phenology (Chen *et al*., 2020; Fu *et al*., 2014; Gao *et al*., 2023; He *et al*., 2024). Among these, the study by Zani *et al*. (2020) was particularly influential, which proposes that increases in spring and summer productivity due to elevated carbon dioxide, temperature, or light levels drive earlier senescence. It sparked debate over whether results from numerous FACE (Free‐Air Carbon Dioxide Enrichment) experiments were consistent with its findings (Norby, 2021; Zani *et al*., 2021). A first general mechanism governing the timing of the autumn phases has been proposed by Zohner *et al*. (2023), where at first sight contradicting effects of environmental factors on the timing of leaf senescence could be accommodated.

In agricultural science, the integration of phenology into yield and water‐use models (Yang *et al*., 2022) has advanced decision‐support systems and climate‐risk assessments. Thus, the dataset has been pivotal not just for ecological inference but for shaping analytical methods across research fields.

### 3. Contribution to global syntheses and assessments

PEP725 data have played a central role in regional and global syntheses of climate impacts on vegetation. For example, Zohner *et al*. (2023) and Zani *et al*. (2020) used PEP725 to quantify carbon sink limitations from delayed autumn senescence in European forests. Similarly, Yin *et al*. (2024) integrated PEP725 into a hemispheric analysis of phenological trends across land‐use types, exploring human impact patterns on spring and autumn events. W. Wu *et al*. (2021) and Z. Wu *et al*. (2021) used PEP725 data to show that atmospheric brightening, the increase in solar radiation reaching the Earth's surface, mitigates the warming‐induced delays in autumn phenology across Europe.

The dataset is a reference input for pan‐European climate services, including the Copernicus High‐Resolution Vegetation Phenology and Productivity (HR‐VPP, Smets *et al*., 2023) product. PEP725‐derived phenological trends also support public risk assessments, including allergy forecasts and agricultural advisory systems (Wohlfahrt *et al*., 2019; Hughes *et al*., 2024). Although phenology is not formally listed among the current essential climate variables of the Global Climate Observing System (GCOS), earlier reports of the World Meteorological Organization (WMO; e.g. WMO‐TD No. 1484, Koch *et al*., 2007) acknowledged phenology as a key terrestrial climate indicator. As such, PEP725 remains an authoritative reference for Earth observation validation and international climate assessments.

### 4. Capacity‐building and open‐data culture

Beyond its scientific applications, PEP725 has significantly advanced the open‐science culture of plant phenology in Europe. The database is built upon the contributions of national monitoring schemes and thousands of volunteers, embodying the principles of citizen science. By enabling unrestricted access to high‐quality phenological data, PEP725 has lowered the barriers for data‐driven research, particularly for early‐career scientists and researchers in regions lacking monitoring infrastructure.

Studies have documented the advantages of the PEP725 model compared to fragmented national datasets. Its standardized BBCH coding and harmonized site‐year formatting allow cross‐country and cross‐species analysis with minimal preprocessing (Kissling *et al*., 2018). PEP725 has become a model for similar efforts in the USA (USA‐NPN) and Asia (AsiaFlux‐Phen) and is widely cited in data sharing and FAIR principle discussions (Templ, 2025). Requests for data now regularly come from over 25 countries and a wide range of user groups.

### 5. International collaborations

The Pan‐European Phenology (PEP725) project is rooted in a long‐standing tradition of international collaboration among National Meteorological and Hydrological Services (NMHSs), research institutions, and environmental agencies across Europe. Established as a follow‐up to the COST Action 725 (2004–2009), PEP725 today serves as a central platform for coordinated phenological observations, harmonized data provision, and open access dissemination. At the heart of this collaborative framework lies the Expert Team (ET, organized within EUMETNET), a flexible and inclusive body of stakeholders from over 20 countries who contribute observational data and expert knowledge.

The ET supports PEP725 by enabling transnational dialogue on operational, methodological, and scientific challenges in phenological monitoring. It facilitates exchange between members through annual in‐person or hybrid meetings often coordinated together with major scientific events such as the European Geosciences Union (EGU) General Assembly and regular virtual communication. This open and collegial structure, which welcomes not only NMHS delegates but also researchers and practitioners with relevant expertise, ensures that PEP725 remains responsive to the evolving needs of phenology networks across Europe.

## III. Utilization of the PEP725 database in diverse scientific studies

The PEP725 database has been widely adopted across ecological, climatological, agricultural, and remote sensing studies. Table 2 summarises this breadth of use by mapping concrete scientific contributions across multiple research domains.

### 1. Plant physiology

Over 60% of the 140 publications using PEP725 data focus on the physiological mechanisms controlling seasonal timing in plants, making this the most prominent domain of PEP725‐based research. Describing and understanding the plant physiological mechanisms governing the variability of phenological events in time and space is of high relevance. As climate change is expected to continue with its influence on ecosystems, natural and man‐made, specific adaptation measures must be built on reliable projections of future phenological timing.

Chilling, forcing, and photoperiod are thought to constitute the three major environmental cues governing plant phenology in the mid‐ to high latitudes (Ettinger *et al*., 2020). Chilling caused the strongest plant phenological response, photoperiod, the weakest. Increased forcing from climate change is currently exerting the strongest influence on plant phenology. A continued trend toward higher temperatures reduces the effect of autumn and winter chilling, which causes plants to increase their demand for forcing temperatures in spring. Wang *et al*. (2022) estimate that the reduction in chilling due to winter warming from 1951 to 2014 offsets about one‐half of the spring phenological advance caused by the increase in forcing. Similarly, the discrepancy between thermal and phenological seasons can be interpreted as a hint that spring temperature alone cannot explain the observed phenological trends (Fu *et al*., 2023).

The concept of temperature sensitivity as shift of phenological entry date per °C has widely been applied to describe the relationship between temperature change and corresponding shift of phenological entry dates. Because of possible artifacts, when calculating temperature sensitivity, caution has to be exercised interpreting results (Güsewell *et al*., 2017; Ettinger *et al*., 2020). A number of works report a decline of the apparent temperature sensitivity of leaf unfolding based on Ground Phenology and Land Surface Phenology (Fu *et al*., 2015b; He *et al*., 2023; Y. Qiao *et al*., 2023). This could partly be attributable to reduced chilling because of temperature increase and photoperiod limitation when leaf‐unfolding dates move to very early dates in spring.

Zohner *et al*. (2018) demonstrate how photoperiod modulates temperature sensitivity across species and latitudes, revealing interspecific variation in phenological plasticity. Similarly, Liu *et al*. (2020) and Fu *et al*. (2015a) explore the role of chilling requirements and precipitation patterns in controlling the onset of spring growth, including shifts induced by recent warming trends.

The availability of consistent multidecade observations has also supported comparative studies across sites and species. Studies such as Meng *et al*. (2021) and Wang *et al*. (2020) leverage this richness to quantify interspecific variation in the response to climate drivers, linking phenological responsiveness to leaf traits and life‐history strategies.

Temperature sensitivity of leaf longevity (difference of leaf‐out and leaf fall) turned out to be similar (average of six deciduous species +3.32 d/°C) in space and time (+4.43/°C), which is interpreted as long‐term acclimation keeping pace with long‐term genetic adaptation of deciduous trees (Xia *et al*., 2022). He *et al*. (2023) claim a nonsymmetric response of phenological events (leaf onset dates) to cooling vs warming. Phenology reacts stronger in the case of cooling compared to warming. Y. Qiao *et al*. (2023) conclude from their investigations that warming‐induced water stress may drive the observed decline in the responses of tree phenology to growing‐season warming by decelerating photosynthetic productivity.

Meanwhile, other work has highlighted the ecological and physiological relevance of autumn phases – traditionally understudied – such as leaf coloration and senescence, which are now analyzed in relation to carbon allocation and stress responses (Wang *et al*., 2023a,b; C. Wu *et al*., 2022; Zhang *et al*., 2024; Zohner *et al*., 2023).

Taken together, these studies have expanded our physiological understanding of phenology from simple thermal‐sum models to more nuanced frameworks incorporating chilling, photoperiod, soil moisture, and species‐specific traits. While models still struggle to represent this complexity, PEP725 data have laid the foundation for a new generation of mechanistic phenology models.

#### Take‐home message

PEP725 has enabled physiologically grounded, multi‐factorial phenology research at a continental scale, revealing the interplay between temperature, photoperiod, and species traits in both spring and autumn transitions. It now serves as a critical reference dataset for validating physiological hypotheses in field phenology.

### 2. Remote sensing

PEP725 data underpin at least 17 peer‐reviewed remote sensing studies that used its long‐term, ground‐based phenological observations to validate satellite‐derived land surface phenology (LSP) products. These studies critically addressed a key challenge in remote phenology monitoring: the mismatch between satellite‐derived seasonal metrics and actual *in situ* plant responses (commonly termed the LSP‐GP mismatch).

A major application of PEP725 data has been in calibrating and validating vegetation indices such as NDVI and EVI across Europe. For example, C. Wu *et al*. (2022) and J. Wu *et al*. (2022) used PEP725 to assess how well MODIS‐based phenological metrics matched ground observations across multiple climate zones, finding substantial biases in forested and mountainous areas. Similarly, Ferrara *et al*. (2023) integrated PEP725 with Sentinel‐2 imagery to refine retrieval algorithms for detecting green‐up and senescence phases in temperate grasslands. These efforts highlight how PEP725 acts as a spatially rich training dataset to improve the timing precision of remote phenological products.

Beyond calibration, PEP725 also enabled novel investigations into spatial mismatches and sensor limitations. Yin *et al*. (2024) demonstrated that LSP estimates consistently lag ground observations in early‐flushing deciduous species, a bias particularly evident in coarse‐resolution imagery. By cross‐comparing years with strong warming anomalies, studies have confirmed that satellite products often underestimate interannual variability in phenology, a limitation that would have remained undetected without PEP725's historical continuity and site specificity.

#### Take‐home message

PEP725 has become the de facto ground truth reference for remote phenology validation in Europe. It provides essential benchmarks for satellite algorithm development and climate change impact assessments, particularly in forest and alpine ecosystems where satellite signals are noisy or biased. Its integration with Sentinel, MODIS, and new drone‐based systems continues to support both operational monitoring and research‐driven model refinement, reaffirming its central role in next‐generation Earth observation systems.

### 3. Phenological modeling

PEP725 data have contributed to at least 39 peer‐reviewed modelling studies, enabling significant advances in empirical, process‐based, and machine learning phenology models. The dataset's breadth – spanning over 150 years, multiple species, and diverse biogeographical regions – makes it uniquely suited for model calibration, validation, and intercomparison.

Early work such as Olsson & Jönsson (2014) leveraged PEP725 data to construct statistical models predicting phenological phases (e.g. flowering or leaf unfolding) based on temperature, day length, and precipitation. These models revealed species‐specific sensitivities and threshold effects, contributing to the foundational understanding of temperature‐driven phenology across Europe.

More recently, process‐based and hybrid models have emerged that incorporate chilling and forcing dynamics, dormancy release, and photoperiod responses (Xu *et al*., 2018; Wang *et al*., 2022). These models increasingly use PEP725 to benchmark temporal accuracy across species and to assess robustness under extreme conditions, such as spring frost events or heatwaves. For instance, Meier & Bigler (2023) showed how PEP725‐based models helped distinguish between phenotypic plasticity and long‐term adaptation in forest tree phenology under climate change scenarios.

In parallel, machine‐learning approaches have used PEP725 as a gold‐standard training set. Studies such as Gao *et al*. (2023) employed neural networks and ensemble techniques to predict phenological shifts under future climate projections. The diversity and temporal depth of the data improved generalization across ecosystems and enabled more nuanced regional models.

#### Take‐home message

PEP725 data have contributed substantially to the improvement of phenological models. They have supported the development of robust, predictive tools for understanding and forecasting seasonal plant behaviour in response to climate variability. As such, PEP725 functions not only as an observational dataset but also as a modeling benchmark, supporting the harmonization of empirical, mechanistic, and AI‐driven approaches across disciplines and scales.

### 4. Ecosystem studies

PEP725 data have been instrumental in at least 20 peer‐reviewed studies examining ecosystem processes, particularly those linking phenological shifts to biodiversity, trophic interactions, and carbon and nutrient cycling. Its multidecadal, multisite plant phenology records provide the temporal and spatial resolution necessary to analyze ecosystem dynamics in response to climate variability and land‐use change.

Phenology is increasingly recognized as a sensitive indicator of ecosystem functioning. Gottschall *et al*. (2022) and Mellert *et al*. (2018) utilized PEP725 data to investigate interspecific variation in phenophase timing across species communities, revealing cascading effects on interspecies interactions such as pollination or herbivory. These shifts may alter the synchrony of food webs, potentially disrupting trophic linkages.

PEP725 also supports research on phenological contributions to ecosystem services. For instance, Nelson *et al*. (2018) used the dataset to link vegetation green‐up timing with CO_2_ uptake rates, highlighting phenology's role in carbon sequestration. Similarly, Kissling *et al*. (2018) explored phenological diversity as a proxy for ecosystem resilience, demonstrating how earlier or delayed flowering affects resource availability and community stability.

Furthermore, the database feeds into broader initiatives to define essential biodiversity variables (a set of standardized biological measurements that help scientists study, report and manage changes in biodiversity), as described by Stucky *et al*. (2018), who noted that long‐term phenology data such as PEP725 are crucial for tracking ecosystem responses at both national and global scales. This underscores the value of PEP725 not just as a plant science tool but as an ecological observatory embedded in the European environmental research infrastructure.

#### Take‐home message

PEP725 enables cross‐scale ecosystem analyses, from plot‐level biodiversity interactions to continental carbon budgets. By capturing phenological shifts across decades, it provides essential ground data to assess ecosystem integrity, anticipate ecological mismatches, and inform conservation strategies in a changing climate.

### 5. Future climate scenarios

PEP725 data have enabled over a dozen studies focused on modeling future climate scenarios and phenological responses under anticipated warming, water stress, and land‐use shifts. The longitudinal depth and pan‐European coverage of the dataset make it uniquely suited for building, calibrating, and validating phenology‐based climate impact models.

Several studies have used PEP725 to forecast shifts in phenological events under IPCC scenarios. For instance, Wang *et al*. (2022) and Zani *et al*. (2020) explored how spring and autumn phases are projected to change under RCP and SSP pathways, revealing strong nonlinearities and species‐specific responses. These analyses often use both empirical trends and process‐based models that integrate temperature thresholds, chilling requirements, and photoperiod sensitivity – parameters for which PEP725 data provide the necessary empirical backbone.

Wohlfahrt *et al*. (2019) demonstrated that the effect of urban heat islands cannot be used as an analog for temporal future changes in plant phenology. Zhao *et al*. (2021) found a strong uncertainty among the phenological models predicting future spring phenology in a warming world. Similarly Chamberlain *et al*. (2021) demonstrated climate change has reshaped the drivers of late frost risk thereby complicating forecasts to future late frost risk. Their findings, like those of other scenario‐based studies, underscore the growing complexity of phenology–climate relationships as ecosystems shift out of equilibrium with historical climatic baselines.

#### Take‐home message

PEP725 is not only a monitoring tool but also supports the development and testing of plant phenological models. Thus, it enables phenological forecasting under climate change and contributes to risk assessments for agriculture, biodiversity conservation, and land management. Its role in future climate modeling is expected to grow, especially with the increasing integration of phenology into Earth system models and national adaptation planning.

### 6. Agricultural applications

PEP725 data have played an essential role in agricultural science, supporting the optimization of crop calendars, frost risk assessment, and climate adaptation strategies across Europe. Phenological observations, particularly for fruit trees and cereals, have enabled empirical modeling of sowing and harvesting windows, which are critical for yield optimization under changing climatic conditions.

Several studies have demonstrated how historical phenology trends captured in PEP725 can improve predictions of flowering and ripening periods. For example, van Bussel *et al*. (2015) utilized the dataset to assess shifts in phenological timing of wheat and their relationship to climate‐driven productivity trends. Similarly, Fraga *et al*. (2016) incorporated PEP725 phenophases into viticultural models to forecast grapevine development under projected climate change, highlighting increased risk of early‐season frost and the need for cultivar‐specific adaptation measures.

Martínez‐Lüscher *et al*. (2016) leveraged phenological records to study the decoupling of vegetative and reproductive growth phases in vineyards, a phenomenon increasingly observed due to warming. Their findings underscore the growing need to recalibrate traditional agro‐climatic models using long‐term phenology datasets like PEP725.

Potentially increased water stress in a warming climate poses an important risk for agricultural production. Yang *et al*. (2022) found that wine regions with higher water stress during the flowering‐veraison phase tend to show higher potential yield loss. Central European wine regions are inclined to experience slight‐to‐moderate drought conditions along with a negligible‐to‐moderate yield loss, whereas the Mediterranean regions show severe‐to‐extreme drought conditions and substantial yield loss.

Apart from potentially increasing water stress in a warming climate, possible changes of late spring frost risk are of high relevance especially for fruit‐growing areas. Liu *et al*. (2018) found the general lengthening of the growing season in the Northern Hemisphere might lead to more frequent frost days. The overall late frost risk for apple cultures appears to persist in a warming climate and may even increase (Unterberger *et al*., 2018).

#### Take‐home message

PEP725 contributes not only to academic understanding but also to practical applications that enhance agricultural resilience. By linking crop phenology to climatic drivers, PEP725 enables region‐specific optimization of sowing and harvest calendars, assessment of frost and drought risks, and development of climate‐smart agricultural management tools.

### 7. Forest applications

PEP725 data have also been incorporated in forest‐related studies to support research on effects of phenological shifts, forest productivity, and ecosystem responses to climate variability. Long‐term ground observations have become indispensable for modeling phenological responses in trees, calibrating forest growth models, and evaluating forest health under stressors like drought, ozone, and heatwaves. Forests, being perennial and slow to adapt, are particularly sensitive to shifts in phenological timing.

Extreme warmth in autumn not only delays for instance the end of the season, but advances next year's start of greening in broad‐leafed deciduous forests and mixed forests at high latitudes in Europe through the legacy or lagged effect. Additionally, extreme warm spring events increased the productivity of broad‐leafed deciduous forests (Crabbe *et al*., 2016). Warming can also stimulate asynchronous developments of various plant traits, like for instance leaf and cambial phenology (Lin *et al*., 2024). A warming climate increases the drought‐related risks for forests. Wohlgemuth *et al*. (2022) found that uptake of environmental mercury in leaves shows a strong positive relationship with soil water and stomatal conductance, thus making the mercury uptake an indicator of drought. Forest growth models serve as instruments to forecast various forest developments under future climate conditions. Nölte *et al*. (2020) and de Wergifosse *et al*. (2020) were able to improve the results of their forest growth models through implementation of a phenology module.

#### Take‐home message

Long‐term tree phenology from PEP725 enhances forest‐growth models and stress assessments, improving understanding of how temperature, drought, and pollutants shape forest productivity and resilience.

### 8. Phenological databases

PEP725 is not only a vital data source for scientific applications but also a model for how long‐term, high‐resolution phenology data can be structured, curated, and shared. Its open‐access approach, integration of historical records, and harmonized phenophase coding have positioned it at the forefront of database development in plant phenology.

Several studies have directly addressed the value of PEP725 as a phenological infrastructure. Kissling *et al*. (2018) and Stucky *et al*. (2018) highlighted PEP725 as a reference point for creating linked open data resources and for designing the Plant Phenology Ontology (PPO), which enables semantic alignment between global phenology datasets. These efforts allow PEP725 data to be interoperable with other major phenological repositories such as USA‐NPN and NEON, facilitating cross‐continental comparisons and synthesis.

Renner & Chmielewski (2021) explored differences and overlap between the phenological databases maintained by the German Weather Service, the Pan‐European PEP725 project, and the International Phenological Gardens. They mentioned the potential of combining PEP725 data with phenological observations from the IPG network for comparison between the phenology of naturally growing populations and the IPG clones.

Izquierdo‐Verdiguier & Zurita‐Milla (2024) utilized PEP725 as a case study in the development of web‐based data retrieval and visualization tools, showcasing how database design can evolve to meet user needs in terms of accessibility and analytical flexibility. Similarly, Templ (2025) proposed synthetic data generation methods for phenological datasets, using PEP725 to validate imputation strategies while maintaining statistical utility, which are critical steps in advancing open‐data infrastructures.

#### Take‐home message

PEP725 exemplifies best practice in open, standardized, and interoperable phenological data management, providing a model infrastructure that supports FAIR principles, ontology development, and global data integration.

## IV. Future directions: coordinated progress for the phenology community

Despite an increase in scientific output, concerns persist that phenology research struggles to accumulate knowledge in a structured and synergistic way. ‘As research in phenology has been making progress, discrepancies and uncertainties in understanding of the relationship between the timing of plant phenological events and environmental factors still remain unresolved because of substantial variation among species and sites’ (Ettinger *et al*., 2020). Thus, future phenological trends remain uncertain (Güsewell *et al*., 2017). This reflects a broader critique that, despite ample data and increasing technical capacity, the field remains fragmented and lacks coordinated direction.

Our synthesis confirms that while the use of PEP725 has expanded across disciplines, it has not yet led to the kind of shared methodological baselines, reusable tools, or cross‐project integration seen in more mature research domains. However, as a living infrastructure, PEP725 is uniquely well positioned to act as a focal point for overcoming this fragmentation.

Achieving this vision will require stable, long‐term funding and institutional commitment to ensure that the technical infrastructure, coordination, and data stewardship can be maintained and further developed. The main funding mechanisms supporting PEP725 and its sustainability challenges are described in Notes S1.

**Fig. 3:**
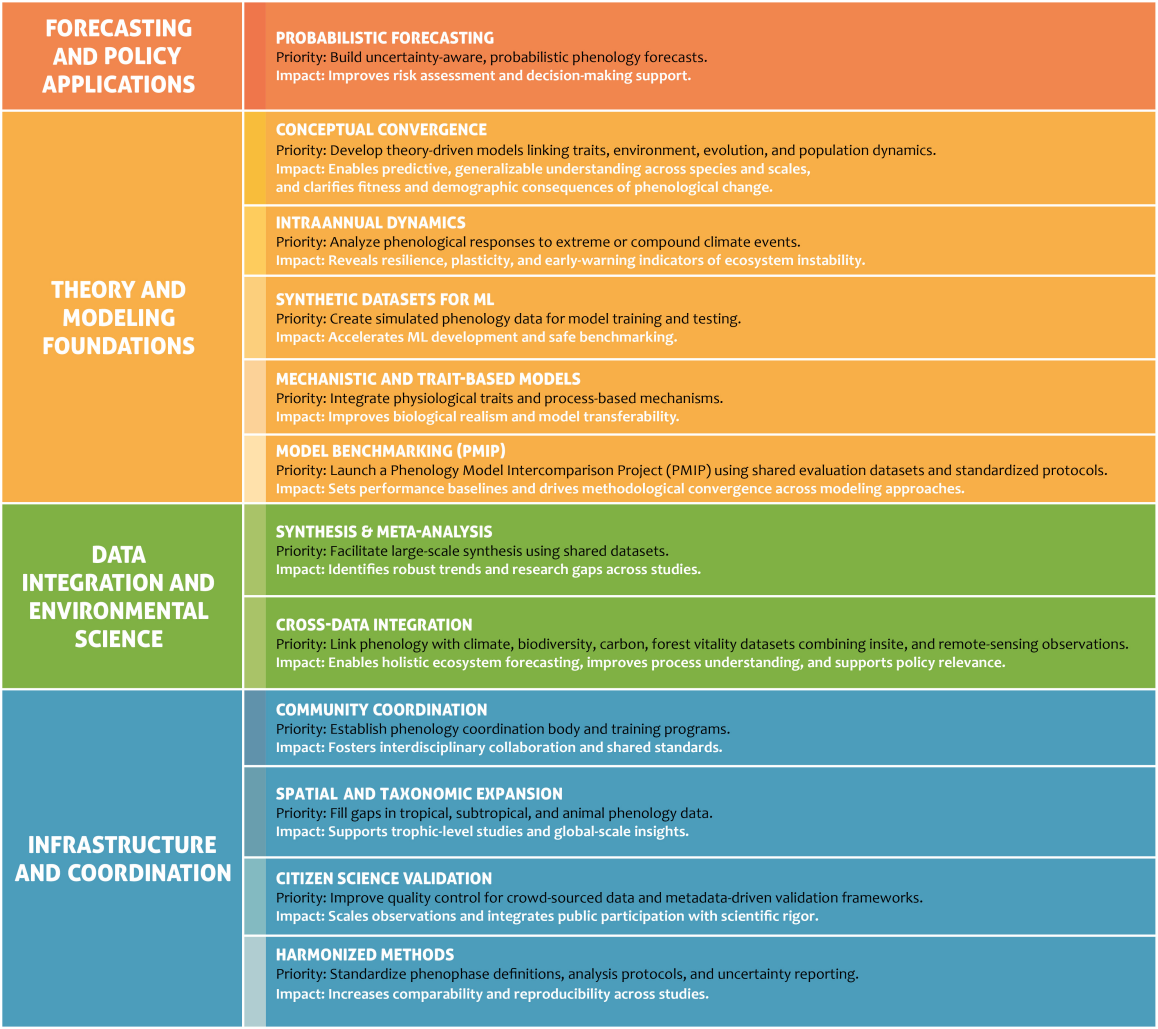
Roadmap for a next‐generation, collaborative phenology science: thematic priorities and time horizons. The schematic summarizes the four major thematic domains guiding the future development of the PEP725 community: infrastructure and coordination, data integration and environmental science, theory and modeling foundations, forecasting and policy applications. Color shading within each thematic block indicates the level of implementation priority, from light (long‐term) to dark (short‐term). ML, machine learning; PMIP, Phenology Model Intercomparison Project; QC, quality control.

As visualized in the color‐coded roadmap (Fig. 3), key priorities are structured along four thematic domains to ensure that phenology research evolves in a more cumulative, impactful, and collaborative direction.

### 1. Infrastructure and coordination

#### Harmonized methodologies and protocols

Despite the standardized internal structure of PEP725, substantial methodological heterogeneity persists across contributing networks, particularly in phenophase definitions, field protocols, species nomenclature, and derivation of phenometric dates. These inconsistencies directly constrain cross‐site synthesis (Tang *et al*., 2016). A coordinated effort toward methodological harmonization is therefore essential. Existing resources provide a solid foundation: the WMO Guidelines for Phenological Observations offer standardized field protocols (Koch, 2010), the PhenObs initiative demonstrates that consistent phenophase definitions can be applied successfully across botanical gardens (Nordt *et al*., 2021), and the Plant Phenology Ontology (PPO) provides a semantic framework to unify event definitions and species information across databases (Stucky *et al*., 2018). Harmonization should also include taxonomic standardization using global nomenclatural backbones such as GBIF. Effective harmonization requires both stringent and flexible components: stringent standards for core variables and metadata to guarantee interoperability, and flexible layers that allow regional or taxon‐specific extensions (Rzanny *et al*., 2024). A conceptual work from Amador *et al*. (2025) further emphasizes that bridging data silos – spanning ground observations, remote sensing, biodiversity repositories, and trait databases – is essential for achieving a unified macrophenological research framework. This broader perspective underlines the need for common semantics and harmonized protocols not only within plant phenology networks but also across related ecological and environmental data infrastructures.

#### Automated data streams and citizen science validation frameworks

To meet future needs, phenology shall expand data acquisition while ensuring high data quality. This is increasingly relevant as citizen science programs and mobile phenology apps generate large volumes of real‐time observations. However, these data streams are often inconsistent in spatial accuracy, observer expertise, and metadata completeness (Kosmala *et al*., 2016; McDonough MacKenzie *et al*., 2017). Recent advances demonstrate that automated quality‐control pipelines can substantially improve reliability. Machine‐learning tools now enable large‐scale automated phenophase annotation from community‐science plant images, achieving high classification accuracy across taxonomic groups (Katal *et al*., 2025). Similarly, machine learning–based anomaly detection has been shown to identify outliers and reporting errors in large observational datasets, offering scalable solutions for cross‐network quality assurance (Hufkens *et al*., 2018b). Beyond technical validation, effective use of citizen‐science data requires structured engagement and error mitigation strategies. A recent synthesis outlines 10 best practices for phenological research – ranging from training observers and documenting protocols to integrating metadata on observer skill and environmental context (Primack *et al*., 2023).

#### Spatial and taxonomic biases

Phenological observations remain disproportionately concentrated in temperate Europe and in a limited set of plant species, while tropical and subtropical regions, as well as many functional groups, are still underrepresented. These uneven sampling patterns can distort estimates of climate sensitivity and trend detection; recent work shows that robust statistical frameworks are needed to map phenoregions and correct spatial biases in large observational datasets (Capinha *et al*., 2024). Taxonomic biases are similarly widespread: biodiversity occurrence records exhibit strong taxonomic and temporal distortions (Melis *et al*., 2025), highlighting the need for harmonized metadata when integrating phenology with broader ecological datasets.

Animal phenology remains especially sparse in continental‐scale databases. Some national networks, such as DWD (Germany) and GeoSphere Austria, collect long‐term observations for taxa like *Apis mellifera*, *Cuculus canorus*, and *Hirundo rustica*, but these datasets are largely unpublished. Cross‐border resources are limited: the EuroBird Portal compiles European bird monitoring data (restricted access), whereas eBird provides a globally accessible platform suitable for linking plant and animal phenology to investigate synchrony and trophic mismatch.

Several initiatives aim to overcome these gaps. The Global Phenological Monitoring Concept (Bruns *et al*., 2003) and GAPON (Global Alliance of Phenological Observation Networks) outline pathways toward global coordination and interoperability, and semantic frameworks such as the Plant Phenology Ontology (PPO; Stucky *et al*., 2018) enable the fusion of heterogeneous networks. Recent multinetwork studies (Ye *et al*., 2022; Qiu *et al*., 2024) demonstrate the scientific value of such integration, revealing cross‐latitude patterns and functional‐group differences in climate sensitivity that cannot be captured within single networks.

Expanding PEP725 through targeted collaborations with data‐sparse regions and linking plant‐based records to emerging animal phenology infrastructures would therefore substantially reduce spatial and taxonomic gaps and support truly ecosystem‐level phenology assessments.

#### Community coordination and training

Institutional fragmentation continues to limit coordinated phenological research. At the international level, the Phenology Commission of the International Biometeorological Society (https://uwm.edu/biometeorology/commissions‐and‐study‐groups/phenologycommission/) and the GLOBE European Phenology Campaign provide frameworks for shared observation goals and early‐detection initiatives. National coordination efforts – such as the Swiss Commission for Phenology and Seasonality (KPS, https://kps.scnat.ch/en), the French TEMPO network (https://tempo.pheno.fr/eng), and the USA National Phenology Network (USA‐NPN, https://www.usanpn.org) demonstrate how structured governance can support standardized monitoring.

USA‐NPN's Local Phenology Leader and Trail Coordinator Certification Programs offer scalable training models, while European initiatives such as the GLOBE Europe Tree Campaign show how cross‐country collaborations can link schools, researchers, and public observers. Culturally grounded programs (e.g. Indigenous phenology curricula) highlight additional pathways for inclusive community engagement.

Integrating these efforts with PEP725 – through a standing Phenology Coordination Group and shared training resources – would improve methodological cohesion, reduce redundancy across networks, and strengthen capacity building within the phenology community.

### 2. Data integration & environmental science

#### Integration with other data streams

To fully realize its potential, phenology must be integrated with complementary environmental data streams. Linking ground observations with climate reanalyses, remote sensing and Earth‐observation products, data from ecosystem and carbon cycle networks, biodiversity and trait datasets as well as dendrochronology records enable cross‐scale analyses and more comprehensive ecosystem assessments. Recent studies illustrate the value of such integration: Chaurasia *et al*. (2024) demonstrate joint modelling of field observations and satellite data in macroecological applications, while Chen & Peñuelas (2025) show how combining *in situ* and remote‐sensing phenology improves detection of climate‐driven trends.

At present, phenological datasets remain siloed from these systems, emphasizing the need for FAIR (Findable, Accessible, Interoperable, Reusable) data principles and developing interoperability standards. Initiatives such as the OSCARS Open and FAIR Integrated Phenology Monitoring System provide concrete models for open infrastructures. Ontology‐based approaches – including the Plant Phenology Ontology (PPO, Stucky *et al*., 2018) and technical guidelines (Templ, 2025) for semantic interoperability and API design – offer a pathway toward harmonized data exchange across networks. Strengthening these connections will allow phenology to contribute more effectively to ecosystem‐level assessments and cross‐sector environmental policy.

#### Synthesis and meta‐analysis

Despite the wealth of available phenological data, synthesis studies remain surprisingly rare (Tang *et al*., 2016). There is a clear need for meta‐analyses and comparative synthesis that draw on multiple datasets. For example, a global meta‐analysis of bird phenology revealed substantially higher temporal variation in urban populations than in nearby rural ones (Capilla‐Lasheras *et al*., 2022), demonstrating the power of cross‐dataset integration for detecting broad ecological patterns. Similarly, a new cross‐taxa meta‐analysis found no consistent demographic consequences of phenological change across species (Godtfredsen *et al*., 2025), illustrating both the potential and the current limitations of phenological synthesis when datasets, metrics, and uncertainty estimates are inconsistent.

To ensure transparency and reproducibility, phenology‐focused syntheses should adopt reporting standards such as PRISMA‐EcoEvo (Preferred Reporting Items for Systematic Reviews and Meta‐Analyses for Ecology and Evolutionary Biology), which provide guidelines for systematic reviews and meta‐analyses in ecology and evolutionary biology (Nakagawa *et al*., 2023). Community‐driven synthesis initiatives – such as data challenges, working groups, or collaborative re‐analysis projects – would accelerate progress by using PEP725 alongside other large‐scale phenology and climate datasets to identify emergent patterns, test shared hypotheses, and benchmark modelling frameworks. To increase visibility and impact, dedicated funding mechanisms and stronger institutional support are required. Funding agencies could explicitly promote cross‐dataset synthesis projects, and major assessment bodies such as the IPCC or GCOS could more prominently feature phenology synthesis products in their reports. Such efforts would consolidate fragmented evidence into actionable insights and elevate phenology's role in global change assessments.

### 3. Theory and modeling foundations

#### Benchmarking and Phenology Model Intercomparison Project (PhenoMIP)

Unlike in climate science – where coordinated multi‐model initiatives such as CMIP (Coupled Model Intercomparison Project) and PMIP (Paleoclimate Modelling Intercomparison Project) provide benchmarks, shared protocols, and reproducible experimental designs, phenology lacks a comparable model intercomparison framework. Early steps toward such benchmarking have emerged (Hufkens *et al*., 2018a; Post *et al*., 2022; Schädel *et al*., 2023).

Community competitions, such as the International Cherry Blossom Forecasting Challenge (George Mason University), provide event‐specific benchmarks where machine learning, statistical, and process‐based models compete on identical training data. These initiatives highlight the benefits of transparent workflows, open datasets, and reproducible evaluation metrics.

What is still missing is a coordinated, European‐scale, multi‐species Phenology Model Intercomparison Project (PhenoMIP). Using PEP725 as a standardized testbed would extend these efforts. Evidence from multimodel ensemble studies in related fields (e.g. Yun *et al*., 2017; Peano *et al*., 2021) shows that ensemble approaches reduce uncertainty and improve robustness, benefits that would carry directly into phenological forecasting.

#### Mechanistic and trait‐based approaches

A growing body of work emphasizes that correlative models alone are insufficient for understanding or forecasting phenological change under shifting climates (Wolkovich *et al*., 2014; Chuine & Régnière, 2017; Piao *et al*., 2019). Mechanistic models, which explicitly represent physiological processes such as dormancy release, chilling accumulation, photoperiod sensitivity, and forcing requirements, provide a more causal and transferable framework. Recent reviews and case studies demonstrate how such models can integrate environmental, demographic, and evolutionary responses (e.g. Jiranek *et al*., 2023) and how process‐based approaches can be extended to both spring and autumn phases across species and sites (Spafford *et al*., 2025).

Despite these advances, empirical links between phenological dynamics and plant functional traits remain underdeveloped. Studies now show that traits such as life form, specific leaf area, bud dormancy depth, or chilling requirements modulate interspecific variation in phenological sensitivity (Jiang *et al*., 2025; Pareja‐Bonilla *et al*., 2025). Integrating PEP725 observations with trait resources such as TRY (https://www.try‐db.org) or forest vitality datasets such as FISE (https://forest.eea.europa.eu/old_countries/hungary/vitality) enables embedding physiological realism directly into predictive models.

#### Synthetic phenology datasets for machine learning and data assimilation

Although PEP725 provides one of the richest ground‐observational phenology datasets globally, it remains sparse across space, time, and species, limiting its suitability for training data‐hungry machine‐learning and data‐assimilation models. In contrast to fields such as genomics or meteorology – where synthetic data and simulation challenges have accelerated methodological innovation – phenology lacks publicly available synthetic or semisynthetic benchmark datasets. Developing synthetic phenology datasets grounded in biological realism (e.g. process‐based simulations of budburst, chilling, forcing, and senescence under climate forcing) would enable training of complex models that currently overfit or fail under sparse observational regimes. Synthetic data would further allow controlled experimentation, stress‐testing of remote‐sensing pipelines, and comparison of competing inference frameworks.

Applications already demonstrate the potential benefits. Semi‐synthetic datasets have supported new approaches in remote phenology extraction, including machine‐learning methods for detecting phenological transitions from multispectral or SIF‐based signals (Fang *et al*., 2023). For image‐based phenology, synthetic augmentation has improved convolutional neural‐network training in citizen‐science contexts (Reeb *et al*., 2022; Stewart *et al*., 2023), showing how simulated data can mitigate class imbalance and observational noise.

Developing an open, community‐standard set of synthetic phenology datasets – analogous to synthetic benchmarks in other environmental sciences – would therefore greatly advance reproducibility, algorithmic comparability, and model generalization across species and biomes.

#### Temporal scaling & intra‐annual dynamics

Most phenology studies focus on single annual events such as first flowering or budburst. Yet many species exhibit pronounced intraannual dynamics – including double leaf‐out events, false springs, or re‐greening, which are increasingly shaped by climate extremes. Recent studies show that subseasonal temperature and precipitation variability strongly modulate these processes in forests and grasslands, affecting community‐level timing and ecosystem functioning (Chen *et al*., 2022; Lorer *et al*., 2025; Matula *et al*., 2023). Methodological advances now allow these dynamics to be resolved with high temporal precision (e.g. Zhang *et al*., 2025). Finally, recent work indicates that intraannual phenological dynamics are mediated by species interactions, which can amplify or buffer the effects of climate extremes (Jankowski *et al*., 2025). Incorporating these subseasonal processes into phenology research will be essential for capturing climate‐sensitivity mechanisms that single annual metrics cannot resolve.

#### Conceptual convergence and theoretical frameworks

While phenological trends are well documented, the field still lacks a unifying theoretical foundation that links traits, environmental drivers, and phylogenetic constraints. However, by shifting the focus from mere pattern description to hypothesis testing and synthesis, the field can build a more predictive and coherent foundation for understanding plant responses across time and space (Wolkovich *et al*., 2014; Piao *et al*., 2019). Emerging frameworks now explicitly address this gap. Hearn & Caetano (2025) introduce a Bayesian, trait–environment model that integrates evolutionary relationships into predictions of phenological timing, providing a formal structure for hypothesis‐driven inference. Such developments illustrate a shift from descriptive pattern reporting toward mechanistic, theory‐oriented models capable of producing generalizable insights across species and regions.

### 4. Forecasting and policy applications

#### Uncertainty propagation and probabilistic forecasting

Real‐world applications of phenological forecasts – from agricultural risk management to biodiversity conservation – require not only accurate predictions but also explicit estimates of forecast uncertainty. For example, crop‐damage assessments increasingly rely on probabilistic predictions of sensitive phenophases during extreme weather events such as hailstorms (Eitzinger *et al*., 2013; Zheng *et al*., 2025). Yet most phenological models still provide point estimates and do not quantify the uncertainty arising from observation error, model structure, or climate inputs, limiting their operational usability under climate variability. Recent methodological advances highlight the need to propagate uncertainty across the entire modeling chain. Frameworks based on Monte Carlo simulation, Bayesian inference, ensemble averaging, and model–data fusion provide tools to partition uncertainty into contributions from field observations, parameter estimation, and climate scenario variance (Migliavacca *et al*., 2012; Taylor & White, 2020; Costafreda‐Aumedes *et al*., 2025). Probabilistic forecasting is increasingly used to support decision‐making. Examples include risk‐based scheduling in agriculture, early‐warning systems for frost and heat damage, and conservation planning under shifting phenological baselines (Ibáñez *et al*., 2010; Wilson *et al*., 2023).

## V. Conclusion

In the 15 years since its establishment, PEP725 has transformed the landscape of phenology research and catalyzed advances in our understanding of plant responses to a changing climate. By adhering to open science principles, PEP725 has proven to be a novel and enduring community resource. It has unified millions of observations into a single, standardized dataset that is leveraged by scientists across disciplines, by educators in the classroom, and by practitioners in agriculture and environmental management. The impacts of this resource are evident in the wealth of high‐impact studies it has supported and the new questions it has enabled researchers to ask. From documenting continent‐wide shifts in seasons to fine‐tuning climate models and satellite algorithms, PEP725 exemplifies how a well‐curated open database can drive both fundamental science and practical applications.

Yet, perhaps the most significant achievement of PEP725 is the community it has built. This includes the network of national meteorological services and volunteers who contribute data, the core team that maintains and improves the infrastructure, and the global user community that analyzes and disseminates findings from the data. Together, this community has demonstrated the power of collaboration and datasharing in plant science. As we look to the future, the continued success of PEP725 will rely on this collective effort. We invite all interested stakeholders to engage with PEP725 – be it by using the data to fuel new research, by contributing additional observations or expertise, or by helping to develop new tools and models that integrate with the platform. There are ample opportunities to get involved: for researchers, this might mean proposing joint analyses or comparative studies; for phenology networks not yet connected, it could mean partnering with PEP725 to broaden the data; for tech‐savvy contributors, perhaps developing open‐source code to enhance data access.

In conclusion, PEP725 stands as a shining example of a community‐driven scientific resource. Its first 15 years have delivered a legacy of improved knowledge and awareness of phenological changes – a legacy built on openness, trust, and shared purpose. By continuing on this path, the next 15 yr promise to further deepen our understanding of phenology in a warming world, inspire innovative solutions in sectors like agriculture and conservation, and foster a vibrant community of phenologists. We encourage readers – whether you are a plant ecologist, a climate scientist, a farmer, or simply a nature enthusiast – to join this endeavor. Use the data, contribute your observations, share your insights, and help us drive phenology science forward. Together, we can ensure that PEP725 remains not just a database, but a living, growing resource at the heart of global phenology research.

## Competing interests

None declared.

## Author contributions

TB and SH conceived the study, designed the research, and developed the overall manuscript structure. SH and RH coordinated data compilation and curation, and RH managed the reference database. TB, UM, and OI designed the figures and tables, while UM and a graphical designer prepared the final figure versions. TB and SH drafted the initial manuscript, and all co‐authors contributed to the revision and approval of the final version. The reviewers provided critical feedback that substantially improved the manuscript.

## Disclaimer

The New Phytologist Foundation remains neutral with regard to jurisdictional claims in maps and in any institutional affiliations.

## Supporting information


**Fig. S1** Number of new user registrations on the PEP725 website.
**Fig. S2** Relative distribution of countries of origin among PEP725 users.
**Methods S1** Workflow for the identification of scientific literature.
**Notes S1** Funding sources and sustainability of the PEP725 database.
**Notes S2** Citation of PEP725‐based studies in the IPCC report.
**Table S1** List of acronyms.
**Table S2** Key characteristics of the contributing institutions to the PEP725 database.
**Table S3** Peer‐reviewed studies using data from the PEP725 database.
Please note: Wiley is not responsible for the content or functionality of any Supporting Information supplied by the authors. Any queries (other than missing material) should be directed to the *New Phytologist* Central Office.
