# Supplementary material for: PEP725: 15 years of driving European and global phenology science
Source: New Phytol. 2026 Jan 22;250(2):717–34. doi: 10.1111/nph.70869 (PMC13001019; doi:10.1111/nph.70869)
Supplement: Supplementary file 1 — Fig. S1 Number of new user registrations on the PEP725 website. Fig. S2 Relative distribution of countries of origin among PEP725 users. Methods S1 Workflow for the identification of scientific literature. Notes S1 Funding sources and sustainability of the PEP725 database. Notes S2 Citation of PEP725‐based studies in the IPCC report. Table S1 List of acronyms. Table S2 Key characteristics of the contributing institutions to the PEP725 database. Table S3 Peer‐reviewed studies using data from the PEP725 database. Please note: Wiley is not responsible for the content or functionality of any Supporting Information supplied by the authors. Any queries (other than missing material) should be directed to the New Phytologist Central Office. [file NPH-250-717-s001.pdf]

New Phytologist Supporting Information

Article title: PEP725: fifteen years of driving European and global phenology science

Authors: Templ, Barbara[1*], Scheifinger, Helfried[2], Ostovary, Isabella[3], Ungersböck, Markus[4], Ressl, Hans[4]

Article acceptance date: 29 November 2025

The following Supporting Information is available for this article:

Fig. S1 Number of new user registrations on the PEP725 website. Data for 2015 are unavailable due to a system failure.

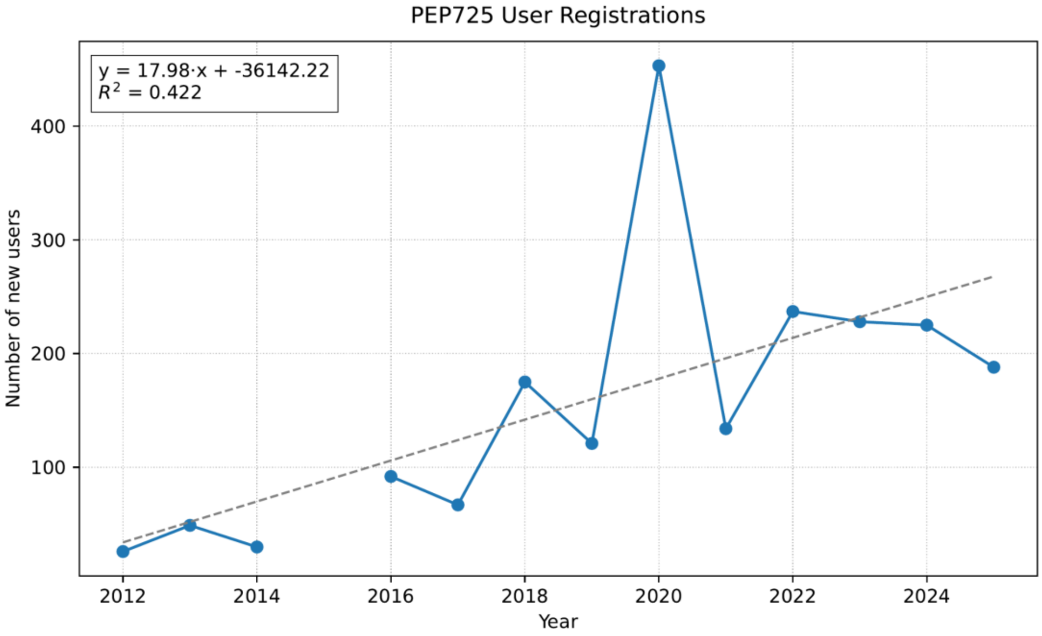

Fig. S2 Relative distribution of countries of origin among PEP725 users. The figure presents the average user distribution for the years 2024 and 2025. China shows by far the highest demand for PEP725 phenological data, representing more than 26% of all users, followed by Spain (11%), Austria (9%) and Germany (8%). At the continental basis, nearly two-thirds of users originate from Europe, while about 30% from Asia. Because inactive accounts are removed after two years, long-term user statistics cannot be derived.

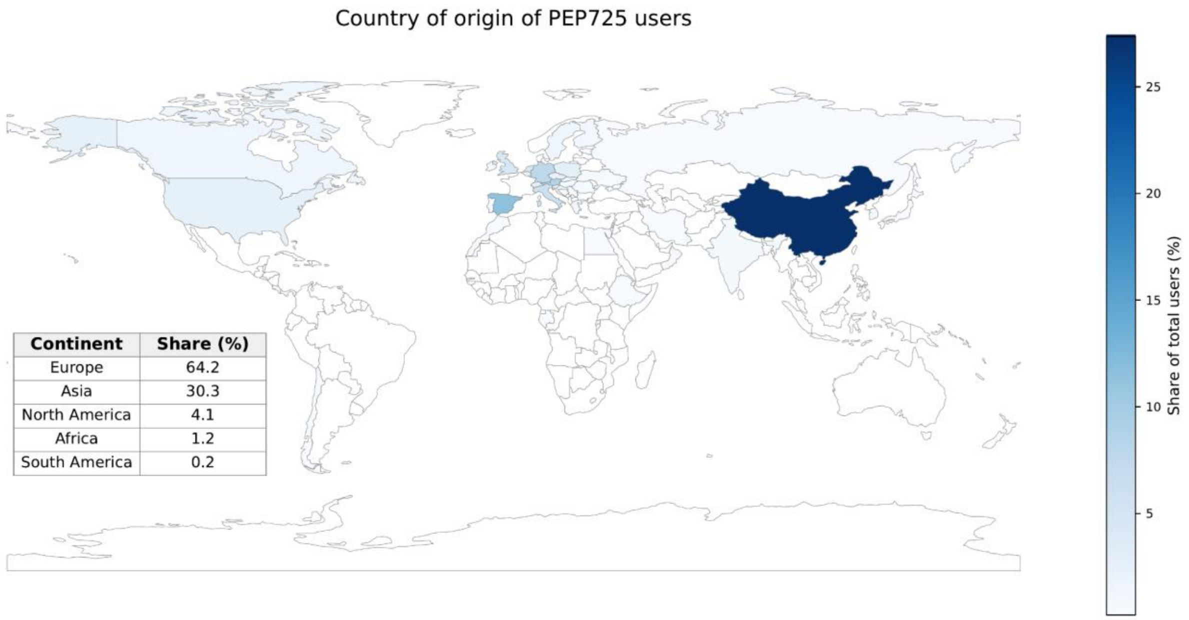

Table S1 List of acronyms.

Acronym	Full term / name	Explanation or context of use
**API**	Application Programming Interface	Standardized interface enabling automated data access to PEP725 and other web services.
**BBCH**	Biologische Bundesanstalt, Bundessortenamt und CHemische Industrie scale	Widely used phenological coding system describing plant growth stages (Meier, 2001).
**COST-725**	European Cooperation in Science and Technology Action 725	Precursor collection of phenological data (2005–2009) that harmonized phenological data across Europe and led to the establishment of PEP725.
**EBV**	Essential Biodiversity Variable	Concept introduced under GEO-BON (Group on Earth Observations Biodiversity Observation Network) for harmonized global biodiversity monitoring; includes phenology as a variable.

**EGU**	European Geosciences Union	The European Geosciences Union (EGU) is the leading organisation for Earth, planetary and space science research in Europe.
**EU**	European Union	Political and funding framework supporting European research infrastructures such as PEP725.
**ET**	EUMETNET Expert Team on Phenology	Technical group coordinating phenological observation methods and metadata standards within EUMETNET.
**EUMETNET**	European Meteorological Network	Association of European National Meteorological and Hydrological Services (NMHSs) fostering collaboration and data exchange.
**EVI**	Enhanced Vegetation Index	Remote-sensing metric used to quantify canopy greenness and seasonal dynamics.
**FACE**	Free-Air CO2 Enrichment	Free-Air CO2 Enrichment, a scientific method for studying the effects of elevated carbon dioxide on plants and ecosystems
**FAIR**	Findable, Accessible, Interoperable and Reusable	Guiding principles for data management and sharing adopted by PEP725.
**FISE**	Forest Information System for Europe	The Forest Information System for Europe (FISE), is a single entry point for data and information on forests in Europe. FISE brings together data, information and knowledge gathered or derived through key forest-related policy drivers.
**GAPON**	Global Alliance of Phenological Observation Networks	Global phenological observation network initiative by the International Society of Biometeorology Phenology Commission (ISB-PC) and the World Meteorological Organization Commission for Agricultural Meteorology (WMO-CAgM)

**GCOS**	Global Climate Observing System	WMO-sponsored programme defining essential climate variables, including phenology-related terrestrial indicators.
**GeoSphere**	GeoSphere Austria	Austrian national geological, geophysical, climatological and meteorological service hosting the PEP725 data infrastructure.
**GP**	Ground Phenology	Ground based phenological observations (as opposed to satellite derived Land Surface Phenology, LSP)
**GPS**	Global Positioning System	Satellite-based positioning system used for georeferencing observation sites.
**HR-VPP**	High-Resolution Vegetation Phenology and Productivity	Copernicus Land Service product providing satellite-based phenology metrics for Europe.
**IPCC**	Intergovernmental Panel on Climate Change	United Nations body assessing climate-change science; PEP725 data contribute to AR6 analyses of phenological responses.
**IPG**	International Phenological Gardens	Network of cloned indicator trees used for long-term phenology monitoring in Europe.
**LSP**	Land Surface Phenology	Seasonal dynamics of vegetation derived from satellite observations.
**ML**	Machine Learning	Multivariate statistical method
**MODIS**	Moderate Resolution Imaging Spectroradiometer	NASA satellite sensor providing global vegetation indices and phenology products.

**NDVI**	Normalized Difference Vegetation Index	Remote-sensing index quantifying greenness, used to estimate phenological transitions.
**NMHS**	National Meteorological and Hydrological Service	National agencies providing meteorological observations integrated in EUMETNET and PEP725.
**PEP725**	Pan European Phenology Database project 725	European data portal providing harmonized in-situ phenological observations from > 25 national networks.
**PMIP**	Paleoclimate Modelling Intercomparison Project	International modelling framework; used here in reference to long-term climate–phenology context.
**PRISMA-EcoEvo**	Preferred Reporting Items for Systematic Reviews and Meta-Analyses for Ecology and Evolutionary Biology	The PRISMA for ecology and evolutionary biology (EcoEvo) provides a 27-item checklist and guidance for reporting systematic reviews and meta-analyses of primary research in ecology and evolutionary biology.
**PPO**	Plant Phenology Ontology	Semantic vocabulary for structuring and exchanging phenological data.
**QC**	Quality Control	An assembly of various methods to evaluate the plausibility of observations
**PRI (sPRIref)**	(Scaled) Photochemical Reflectance Index	The Photochemical Reflectance Index (PRI) is a reflectance measurement, which is sensitive to changes in carotenoid pigments (e.g. xanthophyll pigments) in live foliage.
**RCP**	Representative Concentration Pathway	Climate-forcing scenario used in model simulations of future phenology.
**SIF**	Solar-Induced Fluorescence	SIF vegetation is a red light emitted by plants as a byproduct of photosynthesis. By measuring SIF from space, a plant's photosynthetic activity can be monitored remotely.

**SOS / EOS**	Start of Season / End of Season	Key transition dates in plant phenology derived from observations or remote sensing.
**SSP**	Shared Socio-economic Pathway	Updated scenario framework extending the RCP concept for future climate projections.
**TRY**	Plant Trait Database	TRY is a network of vegetation scientists headed by Future Earth and the Max Planck Institute for Biogeochemistry, providing a global database of curated plant traits.
**USA-NPN**	USA National Phenology Network	American open-data phenology network aligned with FAIR principles.
**VI**	Vegetation Index	A vegetation index (VI) is a numerical value calculated from a satellite's spectral imaging data to monitor vegetation health and density
**WMO**	World Meteorological Organization	UN-specialized agency coordinating international meteorological and climatological activities.
**ZAMG**	Zentralanstalt für Meteorologie und Geodynamik	Former Austrian meteorological service (now part of GeoSphere Austria); initiator and host of the PEP725 project.

Table S2 Key characteristics of the contributing institutions to the PEP725 database. If an institution is listed as "COST-725," the corresponding dataset originates from the COST-725 action and has not been updated since that project ended. This may be due to different circumstances—for example, some national networks discontinued their phenological monitoring (e.g., Belgium), while in other cases data collection was continued or taken over by a different institution (e.g., Poland). Further details on the COST-725 project are provided in Nekovář et al. (2008).

Num-ber	Country	Institution	Begin year	End year	Number of obser-vations	Number of Stations

1	Austria	National Meteorological Service	1775	2025	734487	1573
2	Belgium	COST725	1949	2004	1614	54
3	Bosnia and Herzegovina	National Hydrometeorological Service	1971	2024	9516	6
4	Croatia	National Hydrometeorological Service	1961	2024	40251	11
5	Czech Republic	COST725	1951	2011	15815	135
6	Czech Republic	National Hydrometeorological Service	1961	2024	8028	12
7	Finland	Natural Resources Institute Finland (Luke)	1997	2017	6752	35
8	France	COST725	1963	2005	152	6
9	France	ODS Tela Botanica	2006	2025	22977	1488
10	France	Phénoclim CREA Mont-Blanc	2004	2025	50606	3506
11	France	AgroClim Pheno	1875	1977	6658	3
12	France	Forêt	1872	2024	129299	1058
13	Germany	National Meteorological Service	1951	2024	14098378	6788
14	Germany	School	2014	2018	79	9
15	International	IPG International Phenological Gardens	1962	2009	23492	79

16	Ireland	National Meteorological Service	1966	2024	2716	3
17	Italy	CRI Research and Innovation Centre	1984	2011	181	8
18	Italy	CRA-CMA Agricultural research and experimental organisation	2006	2013	450	62
19	Latvia	COST725	1971	2000	621	8
20	Latvia	University of Latvia	1970	2018	31085	103
21	Lithuania	COST725	1960	2004	27892	30
22	Luxembourg	COST725	1966	2004	183	1
23	Montenegro	National Hydrometeorological Service	1951	2021	13721	7
24	Netherlands	COST725	1868	1968	14610	537
25	North Macedonia	National Hydrometeorological Service	1961	2018	1958	5
26	Norway	COST725	1927	2005	878	4
27	Poland	COST725	1951	1992	6941	13
28	Poland	National Hydrometeorological Service	2007	2018	2682	19
29	Romania	COST725	1982	2005	4220	80

30	Slovakia	National Hydrometeorological Service	2000	2024	109788	76
31	Spain	COST725	1946	2001	1466	15
32	Spain	National Meteorological Service	2013	2020	4696	54
33	Spain	National Meteorological Service	1986	2025	7918	12
34	Sweden	Swedish Agricultural University	2001	2020	2224	5
35	Sweden	National Meteorological Service	1961	2020	35309	14
36	Sweden	COST725	1870	1951	274126	698
37	Sweden	The Swedish National Phenology Network (SLU and SMHI)	1980	2024	134259	1461
38	Switzerland	National Meteorological Service	1951	2024	268412	182
39	UK	Nature's Calendar / Woodland Trust	1950	2005	163440	10509
40	Ukraine	M.M. Gryshko National Botanical Garden NAS of Ukraine	2015	2022	238	1
41	Ukraine	Arboretum Vysokogirny, State Enterprise Nadvirna forestry	2007	2022	96	1

Table S3 Peer-reviewed studies that used data from the PEP725 database (up to 2025).

Basler, D. (2016). Evaluating phenological models for the prediction of leaf-out dates in six temperate tree species across central Europe. *Agricultural and Forest Meteorology*, *217*, 10–21. https://doi.org/10.1016/j.agrformet.2015.11.007

Bórnez, K., Descals, A., Verger, A., & Peñuelas, J. (2020). Land surface phenology from VEGETATION and PROBA-V data. Assessment over deciduous forests. *International Journal of Applied Earth Observation and Geoinformation*, *84*, 101974. https://doi.org/10.1016/j.jag.2019.101974

Bottero, A., Forrester, D. I., Cailleret, M., Kohnle, U., Gessler, A., Michel, D., Bose, A. K., Bauhus, J., Bugmann, H., Cuntz, M., Gillerot, L., Hanewinkel, M., Lévesque, M., Ryder, J., Sainte-Marie, J., Schwarz, J., Yousefpour, R., Zamora-Pereira, J. C., & Rigling, A. (2021). Growth resistance and resilience of mixed silver fir and Norway spruce forests in central Europe: Contrasting responses to mild and severe droughts. *Global Change Biology*, *27*(18), 4403–4419. https://doi.org/10.1111/gcb.15737

Buonaiuto, D. M., Morales-Castilla, I., & Wolkovich, E. M. (2021). Reconciling competing hypotheses regarding flower–leaf sequences in temperate forests for fundamental and global change biology. *New Phytologist*, *229*(3), 1206–1214. https://doi.org/10.1111/nph.16848

Bussel, L. G. J. van, Stehfest, E., Siebert, S., Müller, C., & Ewert, F. (2015). Simulation of the phenological development of wheat and maize at the global scale. *Global Ecology and Biogeography*, *24*(9), 1018–1029. https://doi.org/10.1111/geb.12351

Cailleret, M., Ferretti, M., Gessler, A., Rigling, A., & Schaub, M. (2018). Ozone effects on European forest growth—Towards an integrative approach. *Journal of Ecology*, *106*(4), 1377–1389. https://doi.org/10.1111/1365-2745.12941

Cesaretti, L., Bajocco, S., Corona, P., & Ferrara, C. (2024). *Exploring the Discrepancies between Ground- and Satellite-Based Autumn Phenology: A Comparative Analysis in European Beech Forests* (SSRN Scholarly Paper No. 4854006). Social Science Research Network. https://doi.org/10.2139/ssrn.4854006

Chamberlain, C. J., Cook, B. I., Morales-Castilla, I., & Wolkovich, E. M. (2021). Climate change reshapes the drivers of false spring risk across European trees. *New Phytologist*, *229*(1), 323–334. https://doi.org/10.1111/nph.16851

Chen, L., Hänninen, H., Rossi, S., Smith, N. G., Pau, S., Liu, Z., Feng, G., Gao, J., & Liu, J. (2020). Leaf senescence exhibits stronger climatic responses during warm than during cold autumns. *Nature Climate Change*, *10*(8), 777–780. https://doi.org/10.1038/s41558-020-0820-2

Chen, L., Huang, J., Ma, Q., Hänninen, H., Rossi, S., Piao, S., & Bergeron, Y. (2018). Spring phenology at different altitudes is becoming more uniform under global warming in Europe. *Global Change Biology*, *24*(9), 3969–3975. https://doi.org/10.1111/gcb.14288

Chen, L., Huang, J., Ma, Q., Hänninen, H., Tremblay, F., & Bergeron, Y. (2019). Long-term changes in the impacts of global warming on leaf phenology of four temperate tree species. *Global Change Biology*, *25*(3), 997–1004. https://doi.org/10.1111/gcb.14496

Chen, M., Melaas, E. K., Gray, J. M., Friedl, M. A., & Richardson, A. D. (2016). A new seasonal-deciduous spring phenology submodel in the Community Land Model 4.5: Impacts on carbon and water cycling under future climate scenarios. *Global Change Biology*, *22*(11), 3675–3688. https://doi.org/10.1111/gcb.13326

Chen, X., & Yang, Y. (2020). Observed earlier start of the growing season from middle to high latitudes across the Northern Hemisphere snow-covered landmass for the period 2001–2014. *Environmental Research Letters*, *15*(3), 034042. https://doi.org/10.1088/1748-9326/ab6d39

Cook, B. I., Wolkovich, E. M., Davies, T. J., Ault, T. R., Betancourt, J. L., Allen, J. M., Bolmgren, K., Cleland, E. E., Crimmins, T. M., Kraft, N. J. B., Lancaster, L. T., Mazer, S. J., McCabe, G. J., McGill, B. J., Parmesan, C., Pau, S., Regetz, J., Salamin, N., Schwartz, M. D., & Travers, S. E. (2012). Sensitivity of Spring Phenology to Warming Across Temporal and Spatial Climate Gradients in Two Independent Databases. *Ecosystems*, *15*(8), 1283–1294. https://doi.org/10.1007/s10021-012-9584-5

Crabbe, R. A., Dash, J., Rodriguez-Galiano, V. F., Janous, D., Pavelka, M., & Marek, M. V. (2016). Extreme warm temperatures alter forest phenology and productivity in Europe. *Science of The Total Environment*, *563–564*, 486–495. https://doi.org/10.1016/j.scitotenv.2016.04.124

De Natale, F., Alilla, R., Beltrano, M. C., Dal Monte, G., Epifani, C., Esposito, S., Parisse, B., & Pontrandolfi, A. (2022). Agrometeorological Services for Landscape Agronomy: The Italian Case in the European Context. In D. Rizzo, E. Marraccini, & S. Lardon (Eds.), *Landscape Agronomy: Advances and Challenges of a Territorial Approach to Agricultural Issues* (pp. 27–61). Springer International Publishing. https://doi.org/10.1007/978-3-031-05263-7_2

Delpierre, N., Guillemot, J., Dufrêne, E., Cecchini, S., & Nicolas, M. (2017). Tree phenological ranks repeat from year to year and correlate with growth in temperate deciduous forests. *Agricultural and Forest Meteorology*, *234–235*, 1–10. https://doi.org/10.1016/j.agrformet.2016.12.008

Desai, A. R., Wohlfahrt, G., Zeeman, M. J., Katata, G., Eugster, W., Montagnani, L., Gianelle, D., Mauder, M., & Schmid, H.-P. (2016). Montane ecosystem productivity responds more to global circulation patterns than climatic trends. *Environmental Research Letters*, *11*(2), 024013. https://doi.org/10.1088/1748-9326/11/2/024013

Dolschak, K., Gartner, K., & Berger, T. W. (2019). The impact of rising temperatures on water balance and phenology of European beech (Fagus sylvatica L.) stands. *Modeling Earth Systems and Environment*, *5*(4), 1347–1363. https://doi.org/10.1007/s40808-019-00602-1

Duputié, A., Rutschmann, A., Ronce, O., & Chuine, I. (2015). Phenological plasticity will not help all species adapt to climate change. *Global Change Biology*, *21*(8), 3062–3073. https://doi.org/10.1111/gcb.12914

Ettinger, A. K., Chamberlain, C. J., Morales-Castilla, I., Buonaiuto, D. M., Flynn, D. F. B., Savas, T., Samaha, J. A., & Wolkovich, E. M. (2020). Winter temperatures predominate in spring phenological responses to warming. *Nature Climate Change*, *10*(12), 1137–1142. https://doi.org/10.1038/s41558-020-00917-3

Ferrara, C., Chianucci, F., & Bajocco, S. (2023). On the temporal mismatch between in-situ and satellite-derived spring phenology of European beech forests. *International Journal of Remote Sensing*, *44*(5), 1684–1701. https://doi.org/10.1080/01431161.2023.2189033

Fraga, H., García de Cortázar Atauri, I., Malheiro, A. C., & Santos, J. A. (2016). Modelling climate change impacts on viticultural yield, phenology and stress conditions in Europe. *Global Change Biology*, *22*(11), 3774–3788. https://doi.org/10.1111/gcb.13382

Fu, Y. H., Geng, X., Chen, S., Wu, H., Hao, F., Zhang, X., Wu, Z., Zhang, J., Tang, J., Vitasse, Y., Zohner, C. M., Janssens, I., Stenseth, N. Chr., & Peñuelas, J. (2023). Global warming is increasing the discrepancy between green (actual) and thermal (potential) seasons of temperate trees. *Global Change Biology*, *29*(5), 1377–1389. https://doi.org/10.1111/gcb.16545

Fu, Y. H., Geng, X., Hao, F., Vitasse, Y., Zohner, C. M., Zhang, X., Zhou, X., Yin, G., Peñuelas, J., Piao, S., & Janssens, I. A. (2019). Shortened temperature-relevant period of spring leaf-

out in temperate-zone trees. *Global Change Biology*, *25*(12), 4282–4290. https://doi.org/10.1111/gcb.14782

Fu, Y. H., Piao, S., Op de Beeck, M., Cong, N., Zhao, H., Zhang, Y., Menzel, A., & Janssens, I. A. (2014). Recent spring phenology shifts in western Central Europe based on multiscale observations. *Global Ecology and Biogeography*, *23*(11), 1255–1263. https://doi.org/10.1111/geb.12210

Fu, Y. H., Piao, S., Vitasse, Y., Zhao, H., De Boeck, H. J., Liu, Q., Yang, H., Weber, U., Hänninen, H., & Janssens, I. A. (2015). Increased heat requirement for leaf flushing in temperate woody species over 1980–2012: Effects of chilling, precipitation and insolation. *Global Change Biology*, *21*(7), 2687–2697. https://doi.org/10.1111/gcb.12863

Fu, Y. H., Zhang, X., Piao, S., Hao, F., Geng, X., Vitasse, Y., Zohner, C., Peñuelas, J., & Janssens, I. A. (2019). Daylength helps temperate deciduous trees to leaf-out at the optimal time. *Global Change Biology*, *25*(7), 2410–2418. https://doi.org/10.1111/gcb.14633

Fu, Y. H., Zhao, H., Piao, S., Peaucelle, M., Peng, S., Zhou, G., Ciais, P., Huang, M., Menzel, A., Peñuelas, J., Song, Y., Vitasse, Y., Zeng, Z., & Janssens, I. A. (2015). Declining global warming effects on the phenology of spring leaf unfolding. *Nature*, *526*(7571), 104–107. https://doi.org/10.1038/nature15402

Fu, Y. S. H., Campioli, M., Vitasse, Y., De Boeck, H. J., Van Den Berge, J., AbdElgawad, H., Asard, H., Piao, S., Deckmyn, G., & Janssens, I. A. (2014). Variation in leaf flushing date influences autumnal senescence and next year's flushing date in two temperate tree species. *Proceedings of the National Academy of Sciences*, *111*(20), 7355–7360. https://doi.org/10.1073/pnas.1321727111

Gao, C., Wang, H., & Ge, Q. (2023). Interpretable machine learning algorithms to predict leaf senescence date of deciduous trees. *Agricultural and Forest Meteorology*, *340*, 109623. https://doi.org/10.1016/j.agrformet.2023.109623

Gao, C., Wang, H., Ge, Q., & Dai, J. (2024). Interpreting the influences of multiple factors on forcing requirements of leaf unfolding date by explainable machine learning algorithms. *Ecological Indicators*, *166*, 112402. https://doi.org/10.1016/j.ecolind.2024.112402

Gao, M., Piao, S., Chen, A., Yang, H., Liu, Q., Fu, Y. H., & Janssens, I. A. (2019). Divergent changes in the elevational gradient of vegetation activities over the last 30 years. *Nature Communications*, *10*(1), 2970. https://doi.org/10.1038/s41467-019-11035-w

Gao, X., Richardson, A. D., Friedl, M. A., Moon, M., & Gray, J. M. (2024). Thermal Forcing Versus Chilling? Misspecification of Temperature Controls in Spring Phenology Models. *Global Ecology and Biogeography*, *33*(12), e13932. https://doi.org/10.1111/geb.13932

Geng, X., Fu, Y. H., Hao, F., Zhou, X., Zhang, X., Yin, G., Vitasse, Y., Piao, S., Niu, K., De Boeck, H. J., Menzel, A., & Peñuelas, J. (2020). Climate warming increases spring phenological differences among temperate trees. *Global Change Biology*, *26*(10), 5979–5987. https://doi.org/10.1111/gcb.15301

Geng, X., Fu, Y. H., Piao, S., Hao, F., De Boeck, H. J., Zhang, X., Chen, S., Guo, Y., Prevéy, J. S., Vitasse, Y., Peñuelas, J., Janssens, I. A., & Stenseth, N. Chr. (2022). Higher temperature sensitivity of flowering than leaf-out alters the time between phenophases across temperate tree species. *Global Ecology and Biogeography*, *31*(5), 901–911. https://doi.org/10.1111/geb.13463

Geng, X., Zhang, Y., Fu, Y. H., Hao, F., Janssens, I. A., Peñuelas, J., Piao, S., Tang, J., Wu, Z., Zhang, J., Zhang, X., & Stenseth, N. Chr. (2022). Contrasting phenology responses to climate warming across the northern extra-tropics. *Fundamental Research*, *2*(5), 708–715. https://doi.org/10.1016/j.fmre.2021.11.035

Gonsamo, A., & Chen, J. M. (2016). Circumpolar vegetation dynamics product for global change study. *Remote Sensing of Environment*, *182*, 13–26. https://doi.org/10.1016/j.rse.2016.04.022

Gottschall, F., Cesarz, S., Auge, H., Kovach, K. R., Mori, A. S., Nock, C. A., & Eisenhauer, N. (2022). Spatiotemporal dynamics of abiotic and biotic properties explain biodiversity–ecosystem-functioning relationships. *Ecological Monographs*, *92*(1), e01490. https://doi.org/10.1002/ecm.1490

Gu, H., Qiao, Y., Xi, Z., Rossi, S., Smith, N., Liu, J., & Chen, L. (2022). *Warming-induced increase in carbon uptake leads to earlier spring phenology*. Research Square. https://doi.org/10.21203/rs.3.rs-1390761/v1

Guan, B. T. (2014). Ensemble empirical mode decomposition for analyzing phenological responses to warming. *Agricultural and Forest Meteorology*, *194*, 1–7. https://doi.org/10.1016/j.agrformet.2014.03.010

Hamunyela, E., Verbesselt, J., Roerink, G., & Herold, M. (2013). Trends in Spring Phenology of Western European Deciduous Forests. *Remote Sensing*, *5*(12), Article 12. https://doi.org/10.3390/rs5126159

He, L., Wang, J., Ciais, P., Ballantyne, A., Yu, K., Zhang, W., Xiao, J., Ritter, F., Liu, Z., Wang, X., Li, X., Peng, S., Ma, C., Zhou, C., Li, Z.-L., Xie, Y., & Ye, J.-S. (2023). Non-symmetric responses of leaf onset date to natural warming and cooling in northern ecosystems. *PNAS Nexus*, *2*(9), 308. https://doi.org/10.1093/pnasnexus/pgad308

He, L., Wang, J., Peñuelas, J., Zohner, C. M., Crowther, T. W., Fu, Y., Zhang, W., Xiao, J., Liu, Z., Wang, X., Li, J.-H., Li, X., Peng, S., Xie, Y., Ye, J.-S., Zhou, C., & Li, Z.-L. (2024). Asymmetric temperature effect on leaf senescence and its control on ecosystem productivity. *PNAS Nexus*, *3*(11), pgae477. https://doi.org/10.1093/pnasnexus/pgae477

He, X., Chen, S., Wang, J., Smith, N. G., Rossi, S., Yang, H., Liu, J., & Chen, L. (2021). Delaying effect of humidity on leaf unfolding in Europe. *Science of The Total Environment*, *800*, 149563. https://doi.org/10.1016/j.scitotenv.2021.149563

Hufkens, K., Basler, D., Milliman, T., Melaas, E. K., & Richardson, A. D. (2018). An integrated phenology modelling framework in r. *Methods in Ecology and Evolution*, *9*(5), 1276–1285. https://doi.org/10.1111/2041-210X.12970

Hughes, G. O., Eatherall, A., Bird, M., Blake, J., Branford, P. R., Gebler, S., Lozano, A., Massey, P. A., Reinken, G., Terry, A. S., & Whitworth, E. H. (2024). CropLife Europe Crop Development Database: An open-source, pan-European, harmonized crop development database for use in regulatory pesticide exposure modeling and risk assessment. *Integrated Environmental Assessment and Management*, *20*(4), 1060–1074. https://doi.org/10.1002/ieam.4870

Izquierdo-Verdiguier, E., & Zurita-Milla, R. (2024). A multi-decadal 1 km gridded database of continental-scale spring onset products. *Scientific Data*, *11*(1), 905. https://doi.org/10.1038/s41597-024-03710-5

Jochner, S., Sparks, T. H., Laube, J., & Menzel, A. (2016). Can we detect a nonlinear response to temperature in European plant phenology? *International Journal of Biometeorology*, *60*(10), 1551–1561. https://doi.org/10.1007/s00484-016-1146-7

Kissling, W. D., Walls, R., Bowser, A., Jones, M. O., Kattge, J., Agosti, D., Amengual, J., Basset, A., van Bodegom, P. M., Cornelissen, J. H. C., Denny, E. G., Deudero, S., Egloff, W., Elmendorf, S. C., Alonso García, E., Jones, K. D., Jones, O. R., Lavorel, S., Lear, D., … Guralnick, R. P. (2018). Towards global data products of Essential Biodiversity Variables on species traits. *Nature Ecology & Evolution*, *2*(10), 1531–1540. https://doi.org/10.1038/s41559-018-0667-3

Kowalski, K., Senf, C., Hostert, P., & Pflugmacher, D. (2020). Characterizing spring phenology of temperate broadleaf forests using Landsat and Sentinel-2 time series. *International Journal of Applied Earth Observation and Geoinformation*, *92*, 102172. https://doi.org/10.1016/j.jag.2020.102172

Lapenis, A., Henry, H., Vuille, M., & Mower, J. (2014). Climatic factors controlling plant sensitivity to warming. *Climatic Change*, *122*(4), 723–734. https://doi.org/10.1007/s10584-013-1010-2

Li, D., Stucky, B. J., Baiser, B., & Guralnick, R. (2021). Urbanization delays plant leaf senescence and extends growing season length in cold but not in warm areas of the Northern Hemisphere. *Global Ecology and Biogeography*, *31*(2), 308–320. https://doi.org/10.1111/geb.13429

Li, X., Fan, R., Pan, X., Chen, H., & Ma, Q. (2024). Climate warming advances phenological sequences of *Aesculus hippocastanum*. *Agricultural and Forest Meteorology*, *349*, 109958. https://doi.org/10.1016/j.agrformet.2024.109958

Lian, X., Jiao, L., Zhong, J., Jia, Q., Liu, J., & Liu, Z. (2021). Artificial light pollution inhibits plant phenology advance induced by climate warming. *Environmental Pollution*, *291*, 118110. https://doi.org/10.1016/j.envpol.2021.118110

Lin, S., Wang, H., Dai, J., & Ge, Q. (2024). Spring wood phenology responds more strongly to chilling temperatures than bud phenology in European conifers. *Tree Physiology*, *44*(1), tpad146. https://doi.org/10.1093/treephys/tpad146

Liu, Q., Piao, S., Campioli, M., Gao, M., Fu, Y. H., Wang, K., He, Y., Li, X., & Janssens, I. A. (2020). Modeling leaf senescence of deciduous tree species in Europe. *Global Change Biology*, *26*(7), 4104–4118. https://doi.org/10.1111/gcb.15132

Liu, Q., Piao, S., Janssens, I. A., Fu, Y., Peng, S., Lian, X., Ciais, P., Myneni, R. B., Peñuelas, J., & Wang, T. (2018). Extension of the growing season increases vegetation exposure to frost. *Nature Communications*, *9*(1), 426. https://doi.org/10.1038/s41467-017-02690-y

Liu, Y., Wu, C., Tian, F., Wang, X., Gamon, J. A., Wong, C. Y. S., Zhang, X., Gonsamo, A., & Jassal, R. S. (2022). Modeling plant phenology by MODIS derived photochemical reflectance index (PRI). *Agricultural and Forest Meteorology*, *324*, 109095. https://doi.org/10.1016/j.agrformet.2022.109095

Ma, Q., Hänninen, H., Berninger, F., Li, X., & Huang, J.-G. (2022). Climate warming leads to advanced fruit development period of temperate woody species but divergent changes in its length. *Global Change Biology*, *28*(20), 6021–6032. https://doi.org/10.1111/gcb.16357

Ma, Q., Huang, J.-G., Hänninen, H., & Berninger, F. (2018). Reduced geographical variability in spring phenology of temperate trees with recent warming. *Agricultural and Forest Meteorology*, *256–257*, 526–533. https://doi.org/10.1016/j.agrformet.2018.04.012

Ma, Q., Huang, J.-G., Hänninen, H., & Berninger, F. (2019). Divergent trends in the risk of spring frost damage to trees in Europe with recent warming. *Global Change Biology*, *25*(1), 351–360. https://doi.org/10.1111/gcb.14479

Ma, Q., Huang, J.-G., Hänninen, H., Li, X., & Berninger, F. (2021). Climate warming prolongs the time interval between leaf-out and flowering in temperate trees: Effects of chilling, forcing and photoperiod. *Journal of Ecology*, *109*(3), 1319–1330. https://doi.org/10.1111/1365-2745.13558

Marqués, L., Hufkens, K., Bigler, C., Crowther, T. W., Zohner, C. M., & Stocker, B. D. (2022). Acclimation of phenology relieves leaf longevity constraints in deciduous forests. *Nature Ecology & Evolution*, *7*(2), 198–204. https://doi.org/10.1038/s41559-022-01946-1

Martínez-Lüscher, J., Kizildeniz, T., Vučetić, V., Dai, Z., Luedeling, E., van Leeuwen, C., Gomès, E., Pascual, I., Irigoyen, J. J., Morales, F., & Delrot, S. (2016). Sensitivity of Grapevine Phenology to Water Availability, Temperature and CO2 Concentration. *Frontiers in Environmental Science*, *4*. https://doi.org/10.3389/fenvs.2016.00048

Meier, M., & Bigler, C. (2023). Process-oriented models of autumn leaf phenology: Ways to sound calibration and implications of uncertain projections. *Geoscientific Model Development*, *16*(23), 7171–7201. https://doi.org/10.5194/gmd-16-7171-2023

Meier, M., Bugmann, H., & Bigler, C. (2024). Process-oriented models of leaf senescence are biased towards the mean: Impacts on model performance and future projections. *Global Change Biology*, *30*(1), e17099. https://doi.org/10.1111/gcb.17099

Mellert, K. H., Lenoir, J., Winter, S., Kölling, C., Čarni, A., Dorado-Liñán, I., Gégout, J.-C., Göttlein, A., Hornstein, D., Jantsch, M., Juvan, N., Kolb, E., López-Senespleda, E., Menzel, A., Stojanović, D., Täger, S., Tsiripidis, I., Wohlgemuth, T., & Ewald, J. (2018). Soil water storage appears to compensate for climatic aridity at the xeric margin of European tree species distribution. *European Journal of Forest Research*, *137*(1), 79–92. https://doi.org/10.1007/s10342-017-1092-x

Meng, L., Zhou, Y., Gu, L., Richardson, A. D., Peñuelas, J., Fu, Y., Wang, Y., Asrar, G. R., De Boeck, H. J., Mao, J., Zhang, Y., & Wang, Z. (2021). Photoperiod decelerates the advance of spring phenology of six deciduous tree species under climate warming. *Global Change Biology*, *27*(12), 2914–2927. https://doi.org/10.1111/gcb.15575

Menzel, A., Yuan, Y., Matiu, M., Sparks, T., Scheifinger, H., Gehrig, R., & Estrella, N. (2020). Climate change fingerprints in recent European plant phenology. *Global Change Biology*, *26*(4), 2599–2612. https://doi.org/10.1111/gcb.15000

Nelson, D. B., Ladd, S. N., Schubert, C. J., & Kahmen, A. (2018). Rapid atmospheric transport and large-scale deposition of recently synthesized plant waxes. *Geochimica et Cosmochimica Acta*, *222*, 599–617. https://doi.org/10.1016/j.gca.2017.11.018

Nölte, A., Yousefpour, R., & Hanewinkel, M. (2020). Changes in sessile oak (Quercus petraea) productivity under climate change by improved leaf phenology in the 3-PG model. *Ecological Modelling*, *438*, 109285. https://doi.org/10.1016/j.ecolmodel.2020.109285

Olsson, C., & Jönsson, A. M. (2014). Process-based models not always better than empirical models for simulating budburst of Norway spruce and birch in Europe. *Global Change Biology*, *20*(11), 3492–3507. https://doi.org/10.1111/gcb.12593

Paoli, A., Weladji, R. B., Holand, Ø., & Kumpula, J. (2019). The onset in spring and the end in autumn of the thermal and vegetative growing season affect calving time and reproductive success in reindeer. *Current Zoology*, *66*(2), 123–134. https://doi.org/10.1093/cz/zoz032

Peaucelle, M., Janssens, I. A., Stocker, B. D., Descals Ferrando, A., Fu, Y. H., Molowny-Horas, R., Ciais, P., & Peñuelas, J. (2019). Spatial variance of spring phenology in temperate deciduous forests is constrained by background climatic conditions. *Nature Communications*, *10*(1), 5388. https://doi.org/10.1038/s41467-019-13365-1

Peaucelle, M., Peñuelas, J., & Verbeeck, H. (2022). Accurate phenology analyses require bud traits and energy budgets. *Nature Plants*, *8*(8), 915–922. https://doi.org/10.1038/s41477-022-01209-8

Perez, M. (2024, July). *Utilizing Phenocam Imagery and Convolutional Neural Network for Plant Phenological State Prediction*. University of Twente. https://essay.utwente.nl/101157/

Pfeil, I., Wagner, W., Forkel, M., Dorigo, W., & Vreugdenhil, M. (2020). Does ASCAT observe the spring reactivation in temperate deciduous broadleaf forests? *Remote Sensing of Environment*, *250*, 112042. https://doi.org/10.1016/j.rse.2020.112042

Piao, S., Liu, Q., Chen, A., Janssens, I. A., Fu, Y., Dai, J., Liu, L., Lian, X., Shen, M., & Zhu, X. (2019). Plant phenology and global climate change: Current progresses and challenges. *Global Change Biology*, *25*(6), 1922–1940. https://doi.org/10.1111/gcb.14619

Piao, S., Tan, J., Chen, A., Fu, Y. H., Ciais, P., Liu, Q., Janssens, I. A., Vicca, S., Zeng, Z., Jeong, S.-J., Li, Y., Myneni, R. B., Peng, S., Shen, M., & Peñuelas, J. (2015). Leaf onset in the northern hemisphere triggered by daytime temperature. *Nature Communications*, *6*(1), 6911. https://doi.org/10.1038/ncomms7911

Picornell, A., Smith, M., & Rojo, J. (2023). Climate change related phenological decoupling in species belonging to the Betulaceae family. *International Journal of Biometeorology*, *67*(1), 195–209. https://doi.org/10.1007/s00484-022-02398-9

Pierre, J.-S., Hullé, M., Gauthier, J.-P., & Rispe, C. (2021). Critical windows: A method for detecting lagged variables in ecological time series. *Ecological Informatics*, *61*, 101178. https://doi.org/10.1016/j.ecoinf.2020.101178

Qiao, S., Harrison, S. P., Prentice, I. C., & Wang, H. (2023). Optimality-based modelling of wheat sowing dates globally. *Agricultural Systems*, *206*, 103608. https://doi.org/10.1016/j.agsy.2023.103608

Qiao, Y., Gu, H., Xu, H., Ma, Q., Zhang, X., Yan, Q., Gao, J., Yang, Y., Rossi, S., Smith, N. G., Liu, J., & Chen, L. (2023). Accelerating effects of growing-season warming on tree seasonal activities are progressively disappearing. *Current Biology*, *33*(17), 3625-3633.e3. https://doi.org/10.1016/j.cub.2023.07.030

Qiu, H., Yan, Q., Yang, Y., Huang, X., Wang, J., Luo, J., Peng, L., Bai, G., Zhang, L., Zhang, R., Fu, Y. H., Wu, C., Peñuelas, J., & Chen, L. (2024). Flowering in the Northern Hemisphere is delayed by frost after leaf-out. *Nature Communications*, *15*(1), 9123. https://doi.org/10.1038/s41467-024-53382-3

Renner, S. S., & Chmielewski, F.-M. (2021). The International Phenological Garden network (1959 to 2021): Its 131 gardens, cloned study species, data archiving, and future. *International Journal of Biometeorology*, *66*(1), 35–43. https://doi.org/10.1007/s00484-021-02185-y

Renner, S. S., & Zohner, C. M. (2018). Climate Change and Phenological Mismatch in Trophic Interactions Among Plants, Insects, and Vertebrates. *Annual Review of Ecology, Evolution, and Systematics*, *49*(Volume 49, 2018), 165–182. https://doi.org/10.1146/annurev-ecolsys-110617-062535

Rodriguez-Galiano, V. F., Dash, J., & Atkinson, P. M. (2015). Intercomparison of satellite sensor land surface phenology and ground phenology in Europe. *Geophysical Research Letters*, *42*(7), 2253–2260. https://doi.org/10.1002/2015GL063586

Román, M. O., Justice, C., Paynter, I., Boucher, P. B., Devadiga, S., Endsley, A., Erb, A., Friedl, M., Gao, H., Giglio, L., Gray, J. M., Hall, D., Hulley, G., Kimball, J., Knyazikhin, Y., Lyapustin, A., Myneni, R. B., Noojipady, P., Pu, J., … Wolfe, R. (2024). Continuity between NASA MODIS Collection 6.1 and VIIRS Collection 2 land products. *Remote Sensing of Environment*, *302*, 113963. https://doi.org/10.1016/j.rse.2023.113963

Sakalli, A., & Simpson, D. (2012). Towards the use of dynamic growing seasons in a chemical transport model. *Biogeosciences*, *9*(12), 5161–5179. https://doi.org/10.5194/bg-9-5161-2012

Shen, M., Jiang, N., Peng, D., Rao, Y., Huang, Y., Fu, Y. H., Yang, W., Zhu, X., Cao, R., Chen, X., Chen, J., Miao, C., Wu, C., Wang, T., Liang, E., & Tang, Y. (2020). Can changes in autumn phenology facilitate earlier green-up date of northern vegetation? *Agricultural and Forest Meteorology*, *291*, 108077. https://doi.org/10.1016/j.agrformet.2020.108077

Smets, B., Cai, Z., Eklundh, L., Tian, F., Bonte, K., Van Hoost, R., De Roo, B., Jacobs, T., Camacho, F., Sanchez-Zapero, J., & others. (2023). Copernicus Land Monitoring Service: High resolution vegetation phenology and productivity (HR-VPP), Seasonal Trajectories and VPP parameters. *User Manual*, *2.3*.

Sobrino, J. A., Julien, Y., & Sòria, G. (2013). Phenology Estimation From Meteosat Second Generation Data. *IEEE Journal of Selected Topics in Applied Earth Observations and Remote Sensing*, *6*(3), 1653–1659. IEEE Journal of Selected Topics in Applied Earth Observations and Remote Sensing. https://doi.org/10.1109/JSTARS.2013.2259577

Stemkovski, M., Bell, J. R., Ellwood, E. R., Inouye, B. D., Kobori, H., Lee, S. D., Lloyd-Evans, T., Primack, R. B., Templ, B., & Pearse, W. D. (2023). Disorder or a new order: How climate change affects phenological variability. *Ecology*, *104*(1), e3846. https://doi.org/10.1002/ecy.3846

Stucky, B. J., Guralnick, R., Deck, J., Denny, E. G., Bolmgren, K., & Walls, R. (2018). The Plant Phenology Ontology: A New Informatics Resource for Large-Scale Integration of Plant Phenology Data. *Frontiers in Plant Science*, *9*. https://doi.org/10.3389/fpls.2018.00517

Tian, F., Cai, Z., Jin, H., Hufkens, K., Scheifinger, H., Tagesson, T., Smets, B., Van Hoolst, R., Bonte, K., Ivits, E., Tong, X., Ardö, J., & Eklundh, L. (2021). Calibrating vegetation phenology from Sentinel-2 using eddy covariance, PhenoCam, and PEP725 networks across Europe. *Remote Sensing of Environment*, *260*, 112456. https://doi.org/10.1016/j.rse.2021.112456

Unterberger, C., Brunner, L., Nabernegg, S., Steininger, K. W., Steiner, A. K., Stabentheiner, E., Monschein, S., & Truhetz, H. (2018). Spring frost risk for regional apple production under a warmer climate. *PLOS ONE*, *13*(7), e0200201. https://doi.org/10.1371/journal.pone.0200201

Vaglio Laurin, G., Cotrina-Sanchez, A., Belelli-Marchesini, L., Tomelleri, E., Battipaglia, G., Cocozza, C., Niccoli, F., Kabala, J. P., Gianelle, D., Vescovo, L., Da Ros, L., & Valentini, R. (2024). Comparing ground below-canopy and satellite spectral data for an improved and integrated forest phenology monitoring system. *Ecological Indicators*, *158*, 111328. https://doi.org/10.1016/j.ecolind.2023.111328

Vandvik, V., Halbritter, A. H., & Telford, R. J. (2018). Greening up the mountain. *Proceedings of the National Academy of Sciences*, *115*(5), 833–835. https://doi.org/10.1073/pnas.1721285115

Verger, A., Filella, I., Baret, F., & Peñuelas, J. (2016a). Land surface phenology from SPOT VEGETATION time series. *Revista de Teledetección*, *47*, Article 47. https://doi.org/10.4995/raet.2016.5718

Verger, A., Filella, I., Baret, F., & Peñuelas, J. (2016b). Vegetation baseline phenology from kilometric global LAI satellite products. *Remote Sensing of Environment*, *178*, 1–14. https://doi.org/10.1016/j.rse.2016.02.057

Vitasse, Y., & Basler, D. (2013). What role for photoperiod in the bud burst phenology of European beech. *European Journal of Forest Research*, *132*(1), 1–8. https://doi.org/10.1007/s10342-012-0661-2

Vogel, J. (2022). Drivers of phenological changes in southern Europe. *International Journal of Biometeorology*, *66*(9), 1903–1914. https://doi.org/10.1007/s00484-022-02331-0

Wang, C., Tang, Y., & Chen, J. (2016). Plant phenological synchrony increases under rapid within-spring warming. *Scientific Reports*, *6*(1), 25460. https://doi.org/10.1038/srep25460

Wang, H., Dai, J., Peñuelas, J., Ge, Q., Fu, Y. H., & Wu, C. (2022). Winter warming offsets one half of the spring warming effects on leaf unfolding. *Global Change Biology*, *28*(20), 6033–6049. https://doi.org/10.1111/gcb.16358

Wang, H., Dai, J., Rutishauser, T., Gonsamo, A., Wu, C., & Ge, Q. (2018). Trends and Variability in Temperature Sensitivity of Lilac Flowering Phenology. *Journal of Geophysical Research: Biogeosciences*, *123*(3), 807–817. https://doi.org/10.1002/2017JG004181

Wang, H., Lin, S., Dai, J., & Ge, Q. (2024). Controlled experiments fail to capture plant phenological response to chilling temperature. *Global Ecology and Biogeography*, *33*(10), e13888. https://doi.org/10.1111/geb.13888

Wang, H., Rutishauser, T., Tao, Z., Zhong, S., Ge, Q., & Dai, J. (2017). Impacts of global warming on phenology of spring leaf unfolding remain stable in the long run. *International Journal of Biometeorology*, *61*(2), 287–292. https://doi.org/10.1007/s00484-016-1210-3

Wang, H., Wu, C., Ciais, P., Peñuelas, J., Dai, J., Fu, Y., & Ge, Q. (2020). Overestimation of the effect of climatic warming on spring phenology due to misrepresentation of chilling. *Nature Communications*, *11*(1), 4945. https://doi.org/10.1038/s41467-020-18743-8

Wang, J., & Liu, D. (2023). Larger diurnal temperature range undermined later autumn leaf senescence with warming in Europe. *Global Ecology and Biogeography*, *32*(5), 734–746. https://doi.org/10.1111/geb.13674

Wang, J., Liu, D., Ciais, P., & Peñuelas, J. (2022). Decreasing rainfall frequency contributes to earlier leaf onset in northern ecosystems. *Nature Climate Change*, *12*(4), 386–392. https://doi.org/10.1038/s41558-022-01285-w

Wang, J., Xi, Z., He, X., Chen, S., Rossi, S., Smith, N. G., Liu, J., & Chen, L. (2021). Contrasting temporal variations in responses of leaf unfolding to daytime and nighttime warming. *Global Change Biology*, *27*(20), 5084–5093. https://doi.org/10.1111/gcb.15777

Wang, M., Li, P., Peng, C., Xiao, J., Zhou, X., Luo, Y., & Zhang, C. (2022). Divergent responses of autumn vegetation phenology to climate extremes over northern middle and high latitudes. *Global Ecology and Biogeography*, *31*(11), 2281–2296. https://doi.org/10.1111/geb.13583

Wang, T., Ottlé, C., Peng, S., Janssens, I. A., Lin, X., Poulter, B., Yue, C., & Ciais, P. (2014). The influence of local spring temperature variance on temperature sensitivity of spring phenology. *Global Change Biology*, *20*(5), 1473–1480. https://doi.org/10.1111/gcb.12509

Wang, X., Wu, C., Liu, Y., Peñuelas, J., & Peng, J. (2023). Earlier leaf senescence dates are constrained by soil moisture. *Global Change Biology*, *29*(6), 1557–1573. https://doi.org/10.1111/gcb.16569

Wang, X., Xu, H., Ma, Q., Luo, Y., He, D., Smith, N. G., Rossi, S., & Chen, L. (2023). Chilling and forcing proceed in parallel to regulate spring leaf unfolding in temperate trees. *Global Ecology and Biogeography*, *32*(11), 1914–1927. https://doi.org/10.1111/geb.13740

Wenden, B., Campoy, J. A., Lecourt, J., López Ortega, G., Blanke, M., Radičević, S., Schüller, E., Spornberger, A., Christen, D., Magein, H., Giovannini, D., Campillo, C., Malchev, S., Peris, J. M., Meland, M., Stehr, R., Charlot, G., & Quero-García, J. (2016). A collection of European sweet cherry phenology data for assessing climate change. *Scientific Data*, *3*(1), 160108. https://doi.org/10.1038/sdata.2016.108

Wergifosse, L. de, André, F., Beudez, N., de Coligny, F., Goosse, H., Jonard, F., Ponette, Q., Titeux, H., Vincke, C., & Jonard, M. (2020). HETEROFOR 1.0: A spatially explicit model for exploring the response of structurally complex forests to uncertain future conditions – Part 2: Phenology and water cycle. *Geoscientific Model Development*, *13*(3), 1459–1498. https://doi.org/10.5194/gmd-13-1459-2020

Wohlfahrt, G., Tomelleri, E., & Hammerle, A. (2019). The urban imprint on plant phenology. *Nature Ecology & Evolution*, *3*(12), 1668–1674. https://doi.org/10.1038/s41559-019-1017-9

Wohlgemuth, L., Rautio, P., Ahrends, B., Russ, A., Vesterdal, L., Waldner, P., Timmermann, V., Eickenscheidt, N., Fürst, A., Greve, M., Roskams, P., Thimonier, A., Nicolas, M., Kowalska, A., Ingerslev, M., Merilä, P., Benham, S., Iacoban, C., Hoch, G., … Jiskra, M. (2022). Physiological and climate controls on foliar mercury uptake by European tree species. *Biogeosciences*, *19*(5), 1335–1353. https://doi.org/10.5194/bg-19-1335-2022

Wolkovich, E. M., Cook, B. I., & Davies, T. J. (2014). Progress towards an interdisciplinary science of plant phenology: Building predictions across space, time and species diversity. *New Phytologist*, *201*(4), 1156–1162. https://doi.org/10.1111/nph.12599

Wu, C., Peng, J., Ciais, P., Peñuelas, J., Wang, H., Beguería, S., Andrew Black, T., Jassal, R. S., Zhang, X., Yuan, W., Liang, E., Wang, X., Hua, H., Liu, R., Ju, W., Fu, Y. H., & Ge, Q. (2022). Increased drought effects on the phenology of autumn leaf senescence. *Nature Climate Change*, *12*(10), 943–949. https://doi.org/10.1038/s41558-022-01464-9

Wu, C., Wang, J., Ciais, P., Peñuelas, J., Zhang, X., Sonnentag, O., Tian, F., Wang, X., Wang, H., Liu, R., Fu, Y. H., & Ge, Q. (2021). Widespread decline in winds delayed autumn foliar senescence over high latitudes. *Proceedings of the National Academy of Sciences*, *118*(16), e2015821118. https://doi.org/10.1073/pnas.2015821118

Wu, C., Wang, X., Wang, H., Ciais, P., Peñuelas, J., Myneni, R. B., Desai, A. R., Gough, C. M., Gonsamo, A., Black, A. T., Jassal, R. S., Ju, W., Yuan, W., Fu, Y., Shen, M., Li, S., Liu, R., Chen, J. M., & Ge, Q. (2018). Contrasting responses of autumn-leaf senescence to daytime and night-time warming. *Nature Climate Change*, *8*(12), 1092–1096. https://doi.org/10.1038/s41558-018-0346-z

Wu, D., Gao, J., Wang, P., Yang, J., Ma, Y., Huo, Z., & Yu, Q. (2024). The necessity of coupling the legacy effect with temperature response in crop phenology models. *Global and Planetary Change*, *240*, 104545. https://doi.org/10.1016/j.gloplacha.2024.104545

Wu, J., Chang, Z., Su, Y., Zhang, C., Wu, X., Bi, C., Liu, L., Yang, X., & Li, X. (2022). Identification of the Spring Green-Up Date Derived from Satellite-Based Vegetation Index over a Heterogeneous Ecoregion. *Remote Sensing*, *14*(17), 4349. https://doi.org/10.3390/rs14174349

Wu, W., Sun, Y., Xiao, K., & Xin, Q. (2021). Development of a global annual land surface phenology dataset for 1982–2018 from the AVHRR data by implementing multiple phenology retrieving methods. *International Journal of Applied Earth Observation and Geoinformation*, *103*, 102487. https://doi.org/10.1016/j.jag.2021.102487

Wu, Z., Chen, S., De Boeck, H. J., Stenseth, N. C., Tang, J., Vitasse, Y., Wang, S., Zohner, C., & Fu, Y. H. (2021). Atmospheric brightening counteracts warming-induced delays in autumn phenology of temperate trees in Europe. *Global Ecology and Biogeography*, *30*(12), 2477–2487. https://doi.org/10.1111/geb.13404

Xia, X., Pan, Y., Chang, M., Wu, D., Zhang, X., Xia, J., & Song, K. (2022). Consistent temperature-dependent patterns of leaf lifespan across spatial and temporal gradients for deciduous trees in Europe. *Science of The Total Environment*, *820*, 153175. https://doi.org/10.1016/j.scitotenv.2022.153175

Xu, Y., Wang, H., Ge, Q., Wu, C., & Dai, J. (2018). The strength of flowering–temperature relationship and preseason length affect temperature sensitivity of first flowering date across space. *International Journal of Climatology*, *38*(13), 5030–5036. https://doi.org/10.1002/joc.5713

Yang, C., Menz, C., Fraga, H., Costafreda-Aumedes, S., Leolini, L., Ramos, M. C., Molitor, D., van Leeuwen, C., & Santos, J. A. (2022). Assessing the grapevine crop water stress indicator over the flowering-veraison phase and the potential yield lose rate in important European wine regions. *Agricultural Water Management*, *261*, 107349. https://doi.org/10.1016/j.agwat.2021.107349

Ye, Y., Zhang, X., Shen, Y., Wang, J., Crimmins, T., & Scheifinger, H. (2022). An optimal method for validating satellite-derived land surface phenology using in-situ observations from national phenology networks. *ISPRS Journal of Photogrammetry and Remote Sensing*, *194*, 74–90. https://doi.org/10.1016/j.isprsjprs.2022.09.018

Yin, H., Liu, Q., Liao, X., Ye, H., Li, Y., & Ma, X. (2024). Refined Analysis of Vegetation Phenology Changes and Driving Forces in High Latitude Altitude Regions of the Northern Hemisphere: Insights from High Temporal Resolution MODIS Products. *Remote Sensing*, *16*(10), Article 10. https://doi.org/10.3390/rs16101744

Zani, D., Crowther, T. W., Mo, L., Renner, S. S., & Zohner, C. M. (2020). Increased growing-season productivity drives earlier autumn leaf senescence in temperate trees. *Science*, *370*(6520), 1066–1071. https://doi.org/10.1126/science.abd8911

Zeng, K., Sentinella, A. T., Armitage, C., & Moles, A. T. (2024). Species that require long-day conditions to flower are not advancing their flowering phenology as fast as species without photoperiod requirements. *Annals of Botany*, mcae121. https://doi.org/10.1093/aob/mcae121

Zhang, H., Chuine, I., Regnier, P., Ciais, P., & Yuan, W. (2022). Deciphering the multiple effects of climate warming on the temporal shift of leaf unfolding. *Nature Climate Change*, *12*(2), 193–199. https://doi.org/10.1038/s41558-021-01261-w

Zhang, X., Ye, Y., & Tran, K. (2023). *Discrepancy and linkage of Satellite-derived Land Surface Phenology with in-situ Observations from National Phenology Networks and PhenoCam Networks* (Nos. EGU23-9227). EGU23. Copernicus Meetings. https://doi.org/10.5194/egusphere-egu23-9227

Zhang, Y., Hong, S., Peñuelas, J., Xu, H., Wang, K., Zhang, Y., Lian, X., & Piao, S. (2024). Weakened connection between spring leaf-out and autumn senescence in the Northern Hemisphere. *Global Change Biology*, *30*(7), e17429. https://doi.org/10.1111/gcb.17429

Zhao, H., Fu, Y. H., Wang, X., Zhang, Y., Liu, Y., & Janssens, I. A. (2021). Diverging models introduce large uncertainty in future climate warming impact on spring phenology of

temperate deciduous trees. *Science of The Total Environment*, *757*, 143903. https://doi.org/10.1016/j.scitotenv.2020.143903

Zheng, X., Zhao, W., Zhu, Z., Wang, Z., Zheng, Y., & Li, D. (2024). Characterization and Evaluation of Global Solar-Induced Chlorophyll Fluorescence Products: Estimation of Gross Primary Productivity and Phenology. *Journal of Remote Sensing*, *4*, 0173. https://doi.org/10.34133/remotesensing.0173

Zohner, C. M., Mirzagholi, L., Renner, S. S., Mo, L., Rebindaine, D., Bucher, R., Palouš, D., Vitasse, Y., Fu, Y. H., Stocker, B. D., & Crowther, T. W. (2023). Effect of climate warming on the timing of autumn leaf senescence reverses after the summer solstice. *Science*, *381*(6653). https://doi.org/10.1126/science.adf5098

Zohner, C. M., Mo, L., Pugh, T. A. M., Bastin, J.-F., & Crowther, T. W. (2020). Interactive climate factors restrict future increases in spring productivity of temperate and boreal trees. *Global Change Biology*, *26*(7), 4042–4055. https://doi.org/10.1111/gcb.15098

Zohner, C. M., Mo, L., & Renner, S. S. (2018). Global warming reduces leaf-out and flowering synchrony among individuals. *eLife*, *7*(30418152), e40214.

Methods S1 Workflow for the systematic identification of scientific literature

To quantify and review the scientific impact and use of PEP725, we conducted a systematic literature search in *Google Scholar* (https://scholar.google.com/ , accessed in May 2025) using the keyword "PEP725 phenology" in the title, abstract, author keywords, or funding acknowledgment fields. All retrieved records were manually checked to verify that PEP725 data were used in the study; duplicates and false positives (e.g., unrelated uses of the abbreviation "PEP") were removed. The resulting dataset comprised approximately 140 peer-reviewed publications that explicitly used PEP725 data.

For comparison, the foundational PEP725 description paper (*Templ et al., 2018;* DOI: 10.1007/s00484-018-1512-8) has been cited ~235 times according to Google Scholar, but not all of these citations involve direct use of the database, as many refer to it for contextual or methodological background. Thus, the count of 140 papers should be regarded as a conservative estimate of actual data use. Not all authors cite PEP725 consistently or report publications to the database administrators as encouraged on the PEP725 website, and some may use the data without explicit acknowledgment.

These publications were categorized by research domain (e.g., phenological modelling, remote sensing, ecosystem studies) and form the basis of the examples summarized in Sections 2 and 3.

Notes S1 Overview of the funding sources and measures ensuring the sustainability of the PEP725 database.

The initial establishment of PEP725 was made possible through the COST Action 725 (2004–2009; https://www.cost.eu/actions/725/, titled: Establishing a European Phenological Data Platform for Climatological Applications), which provided the first European framework for coordinating national phenological networks. Since the end of the COST Action, operational continuity has been guaranteed by GeoSphere Austria. Data management, software development and coordinating activities have been supported by EUMETNET (The European Meteorological Network; https://eumetnet.eu/), which ensures institutional embedding within the meteorological community. In-kind contributions from GeoSphere Austria supplement the EUMETNET funding. Apart from these core supports, the individual PEP725 members and contributors rely solely on their own funding for the operation of their networks. Establishing a stable, multi-annual funding structure, for example through European research-infrastructure or environmental-monitoring frameworks would secure the sustainability and further development of this unique transnational resource.

Notes S2 Citation of PEP725-based studies in the IPCC report

Phenology is referenced 226 times in *Climate Change 2022: impacts, Adaptation and Vulnerability* (IPCC AR6, Working Group II). It plays a central role in the assessment, particularly in discussions of climate-change impacts across ecosystems.
Below we provide the list of IPCC WGII chapters that cite studies based on PEP725 data:

2.4.2.4 Observed Phenological Responses to Climate Change: Piao et al. (2019), Menzel et al. (2020)
2.4.2.5. Observed Complex Phenological and Range Shift Responses: Ettinger et al. (2020)
Table 2.2 Menzel et al. (2020)
Box 5.2: Case Study: Wine: Martinez-Lüscher et al (2016)
Chapter 13.3.1.2 Projected Risks for Terrestrial and Freshwater Ecosystems: Wu et al (2018)
Wang H et al (2020): erroneously this work has been cited in the IPCC text, but is not in the references section.

Ettinger, A. K., Chamberlain, C. J., Morales-Castilla, I., Buonaiuto, D. M., Flynn, D. F. B., Savas, T., Samaha, J. A., & Wolkovich, E. M. (2020). Winter temperatures predominate in spring phenological responses to warming. Nature Climate Change, 1–6.

Martínez-Lüscher, J., Kizildeniz, T., Vučetić, V., Dai, Z., Luedeling, E., van Leeuwen, C., Gomès, E., Pascual, I., Irigoyen, J. J., Morales, F., & Delrot, S. (2016). Sensitivity of Grapevine Phenology to Water Availability, Temperature and CO2 Concentration. Frontiers in Environmental Science, 4, 48. https://doi.org/10.3389/fenvs.2016.00048

Menzel, A., Yuan, Y., Matiu, M., Sparks, T., Scheifinger, H., Gehrig, R., & Estrella, N. (2020).

Climate change fingerprints in recent European plant phenology. Global Change Biology, 26(4), 2599–2612. https://doi.org/DOI: 10.1111/gcb.15000

Piao, S., Liu, Q., Chen, A., Janssens, I. A., Fu, Y., Dai, J., Liu, L., Lian, X., Shen, M., & Zhu, X. (2019). Plant phenology and global climate change: Current progresses and challenges. Global Change Biology, 25(6), 1922–1940. https://doi.org/10.1111/gcb.14619

Wang, H., Wu, C., Ciais, P., Penuelas, J., Dai, J., Fu, Y., & Ge, Q. (2020). Overestimation of the effect of climatic warming on spring phenology due to misrepresentation of chilling. Nature Communications, 11(1), 1–9.

Wu, C., Wang, X., Wang, H., Ciais, P., Peñuelas, J., Myneni, R. B., Desai, A. R., Gough, C. M., Gonsamo, A., Black, A. T., Jassal, R. S., Ju, W., Yuan, W., Fu, Y., Shen, M., Li, S., Liu, R., Chen, J. M., & Ge, Q. (2018). Contrasting responses of autumn-leaf senescence to daytime and night-time warming. Nature Climate Change, 8(12), 1092–1096.
